# Detection of fusion events by RNA sequencing in FFPE versus freshly frozen colorectal cancer tissue samples

**DOI:** 10.3389/fmolb.2024.1448792

**Published:** 2025-01-21

**Authors:** Maxim Sorokin, Vladimir Lyadov, Maria Suntsova, Marat Garipov, Anna Semenova, Natalia Popova, Egor Guguchkin, Rustam Heydarov, Marianna Zolotovskaia, Xiaowen Zhao, Qing Yan, Ye Wang, Evgeny Karpulevich, Anton Buzdin

**Affiliations:** ^1^ OmicsWay Corp., Covina, CA, United States; ^2^ PathoBiology Group, European Organization for Research and Treatment of Cancer (EORTC), Brussels, Belgium; ^3^ Institute of Personalized Oncology, I.M. Sechenov First Moscow State Medical University, Moscow, Russia; ^4^ Moscow Center for Advanced Studies, Moscow, Russia; ^5^ Moscow State Budgetary Healthcare Institution “Moscow City Oncological Hospital N1, Moscow Healthcare Department”, Moscow, Russia; ^6^ Federal State Budgetary Educational Institution of Further Professional Education “Russian Medical Academy of Continuous Professional Education” of the Ministry of Healthcare of the Russian Federation, Moscow, Russia; ^7^ Novokuznetsk State Institute for Advanced Training of Physicians – Branch of RMACPE, Novokuznetsk, Russia; ^8^ Institute for System Programming of RAS, Moscow, Russia; ^9^ Core lab, Qingdao Central Hospital, University of Health and Rehabilitation Sciences, Qingdao, China; ^10^ Group for Genomic Regulation of Cell Signaling Systems, Shemyakin-Ovchinnikov Institute of Bioorganic Chemistry, Moscow, Russia; ^11^ World-Class Research Center “Digital Biodesign and Personalized Healthcare”, Sechenov First Moscow State Medical University, Moscow, Russia

**Keywords:** colorectal cancer, formalin-fixed paraffin-embedded tumor tissue samples, FFPE, RNA sequencing, RNAseq, new cancer fusion genes, chimeric transcripts, detection of gene rearrangements

## Abstract

Gene fusion events result in chimeric proteins that are frequently found in human cancers. Specific targeted therapies are available for several types of cancer fusions including receptor tyrosine kinase gene moieties. RNA sequencing (RNAseq) can directly be used for detection of gene rearrangements in a single test, along with multiple additional biomarkers. However, tumor biosamples are usually formalin-fixed paraffin-embedded (FFPE) tissue blocks where RNA is heavily degraded, which in theory may result in decreased efficiency of fusion detection. Here, for the first time, we compared the efficacy of gene fusion detection by RNAseq for matched pairs of freshly frozen in RNA stabilizing solution (FF) and FFPE tumor tissue samples obtained from 29 human colorectal cancer patients. We detected no statistically significant difference in the number of chimeric transcripts in FFPE and FF RNAseq profiles. The known fusion *KANSL1-ARL17A/B* occurred with a high frequency in 69% of the patients. We also detected 93 new fusion genes not mentioned in the literature or listed in the ChimerSeq database. Among them, 11 were found in two or more patients, suggesting their potential role in carcinogenesis. Most of the fusions detected most probably represented read-through, microdeletion or local duplication events. Finally, in one patient, we detected a potentially clinically actionable in-frame fusion of *LRRFIP2* and *ALK* genes not previously described in colorectal cancer with an intact tyrosine kinase domain that can be potentially targeted by ALK inhibitors.

## Introduction

### Clinical relevance of fusion genes

Fusion genes are frequently found in cancer cell genomes (Li et al., 2023; Sorokin et al., 2022). Some types of oncogenic fusions, especially those involving receptor tyrosine kinase (RTK) genes, are considered clinically applicable because they can be targeted by specific, clinically approved therapeutic agents (Sorokin et al., 2022). In most cases, the role of RTK fusion partner genes is to drive RTK moiety expression at abnormally high levels (Sorokin et al., 2022). This leads to a significant enhancement of proliferation and survival signaling, which promotes tumor development (Schubert et al., 2023; Shreenivas et al., 2023). In turn, relevant RTK activities can be detected, targeted and inhibited by specific drugs. For example, the first-generation ALK inhibitor crizotinib, as well as second- and third-generation drugs such as brigatinib, lorlatinib, alectinib, and ceritinib, have been included in guidelines for the treatment of lung cancer patients with *ALK* gene fusions (Wu et al., 2016). In addition, crizotinib is also approved for the treatment of *ROS1* fusion-positive cancers (Shaw et al., 2014). Entrectinib and larotrectinib are used to treat *NTRK* family fusion-positive solid tumors, marking the first indication for use in cancer based on the detection of a specific type of gene fusion (Doebele et al., 2020; Drilon et al., 2018). The presence of *FGFR2* gene fusion in cholangiocarcinoma is an indication for the administration of infigratinib (Javle et al., 2021) or pemigatinib (Walden et al., 2022). Erdafitinib has been approved for the treatment of urothelial carcinomas with *FGFR2* or *FGFR3* fusion (Loriot et al., 2019). Finally, selpercatinib and pralcetinib are effective in the treatment of solid tumors with *RET* gene rearrangement (Subbiah et al., 2022a; Subbiah et al., 2022b). In addition, many oncogenic fusions are associated with prognosis or may serve as diagnostic biomarkers (Haley et al., 2021; Huang et al., 2023; Zhu et al., 2019). Thus, reliable detection of gene fusions is a high priority in modern cancer treatment.

### Detection of fusion genes

Oncogenic fusion events can be detected with varying degrees of efficiency by whole genome or target DNA sequencing, reverse transcription PCR, immunohistochemistry, or fluorescence *in situ* hybridization (FISH) (Sorokin et al., 2022). Alternatively, these events can be directly detected by analyzing RNA sequencing data by identifying fragments of the corresponding chimeric transcripts (Dorney et al., 2023). RNA analysis offers the advantage of detecting multiple cancer biomarkers in a single test. Indeed, RNA sequencing results can be used to determine tumor mutational burden (Sorokin et al., 2021), assess the status of key immunohistochemistry biomarkers (Sorokin et al., 2020a), evaluate microsatellite instability, measure the expression of molecular targets of anticancer drugs (Buzdin et al., 2020), and interrogate various clinically relevant gene signatures (Lazar et al., 2023; Sorokin et al., 2020b).

Several bioinformatic tools have been developed to detect fused transcripts in RNA sequencing data (Haas et al., 2019). However, there is a certain degree of discrepancy between different such tools (Hafstað et al., 2023). Most of these tools have been tested on fresh tissue samples, which allows the isolation and sequencing of long, high-quality RNA molecules. Although fresh tumor tissue is undoubtedly favorable for nucleic acid molecular analysis, cancer biomaterials are mostly stored as formalin-fixed, paraffin-embedded (FFPE) tissue blocks where RNA undergoes severe degradation, resulting in shorter RNA sequencing reads (Suntsova et al., 2019).

Despite these theoretical considerations, to the best of our knowledge, no study has yet been published that directly compares the efficiency of fusion gene detection in fresh tissue samples compared to FFPE samples. Here, we performed such an analysis for the first time using RNA sequencing of libraries created from matched FFPE biosamples and RNA-stabilized fresh-frozen (FF) colorectal cancer tissues obtained from the same 29 human patients.

## Materials and methods

### Patient enrollment and sample collection

Primary colorectal cancer patients were enrolled in this study. All patients underwent surgical removal of their tumor tissue. For each patient, the tumor tissue was either immediately placed into RNAlater stabilizing solution (Ambion) and stored at −70°C, or fixed in formalin and subsequently embedded into a paraffin (FFPE) block. Since the duration of fixation can be a defining feature for identifying the fusion genes, formalin fixation time for all FFPE samples was 16 h according to the previous protocol (Cappello et al., 2022). Patient inclusion criteria included an age range of 18–75 years and histologically confirmed colorectal cancer.

### RNAseq library preparation and sequencing

RNA was extracted from FFPE slices or RNA-stabilized solutions using the QIAGEN RNeasy Kit, adhering to the manufacturer’s protocol. Library construction and ribosomal RNA depletion were performed using the KAPA RNA Hyper with rRNA Erase (HMR only) kit. To multiplex samples in one sequencing run, different adaptors were utilized. Library concentrations were measured using the Qubit dsDNA HS Assay kit (Life Technologies), and quality was assessed with the Agilent Tapestation (Agilent). RNA sequencing was conducted on the Genolab M engine for paired-end sequencing with a read length of 75 bp.

### RNAseq data processing

RNAseq FASTQ files were processed using the STAR aligner (Dobin et al., 2013) in “GeneCounts” mode, with the Ensembl human transcriptome annotation (Build version GRCh38, transcript annotation GRCh38.89) as a reference. Quantile normalization (Bolstad, 2017) was applied for gene expression clustering and PCA analyses. Cancer fusion transcripts were detected using the STAR-Fusion software (Haas et al., 2019). Identified putative fusion candidates were included in downstream analysis only if they passed specific thresholds, with either a JunctionReadCount greater than 1 or a SpanningFragCount greater than 1.

### Statistics and data visualization

The results were visualized using the R packages ggplot2 and ggpubr. Principal component analysis (PCA) was performed using the prcomp function in R. The Student’s T-test was employed to compare differences between the means, and Spearman’s Rho was calculated for pairwise correlation analysis.

## Results

### Patient enrollment and tumor profiling

In this prospective study, we enrolled 29 patients with histologically confirmed primary colorectal cancer, comprising 17 male (age range 59–84 years, mean age 70 years) and 12 female (age range 62–85 years, mean age 72.5 years) patients. Post-operative tumor tissue specimens were either freshly frozen in RNAlater (FF) or available as formalin-fixed paraffin-embedded (FFPE) blocks. Both types of materials underwent paired-end RNA sequencing with a 75 bp read length. On average, each sample yielded 15 million raw sequencing reads. We employed the STAR-Fusion software to detect chimeric transcripts in the RNAseq profiles and used the ChimerDB database (Jang et al., 2020), the Mitelman Database (https://mitelmandatabase.isb-cgc.org), and PubMed searches with fusion-forming gene IDs to classify fusions as new or previously published. According to the criteria previously deduced for finding cancer gene fusions in FFPE reads (Rabushko et al., 2022), only chimeric transcripts supported by at least two non-duplicated paired reads were considered for further analysis. This data filtering setting, adapted from our previous research, allowed for the identification of novel and known chimeric transcripts in FFPE RNAseq data with nearly 100% specificity, as confirmed by reverse transcription PCR followed by Sanger sequencing of the resulting products (Rabushko et al., 2022).

### Fusion transcript detection and analysis

**FIGURE 1:**
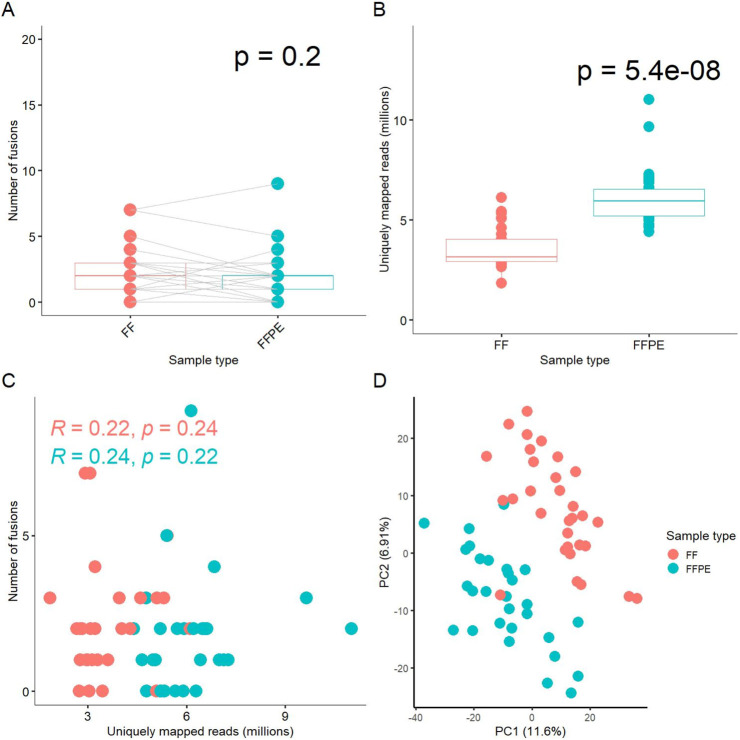
Comparison of experimental FF and FFPE paired gene expression profiles. **(A)** Box-plot for number of fusion transcripts detected in FF versus matched FFPE samples. **(B)** Box-plot for number of uniquely mapped reads in sequenced FF and FFPE libraries. **(C)** Scatterplot for relationship between the number of fusion transcripts detected and the number of uniquely mapped reads in the respective libraries. **(D)** Principal component analysis (PCA) of log-transformed gene expression levels (TPM) in FF and FFPE libraries.

In this study, only one patient’s tumor exhibited the same fusion transcripts in both fresh frozen (FF) and formalin-fixed paraffin-embedded (FFPE) tissue samples; in the remaining cases, the outputs from FF and FFPE paired samples differed (Supplementary Table S1). In total, we identified 113 fusion transcripts, of which 69 included fragments of protein-coding genes and 44 involved fusions of non-coding RNAs (Supplementary Table S1). We detected at least one common fusion transcript in 17 out of 29 cases (59%) in the paired FF/FFPE samples. In 13 cases (45%), the number of detected fusions in FF samples was higher than in the FFPE tumor tissue blocks, while in 10 cases (34%), the number of FFPE fusions was higher. Overall, there was no statistically significant difference in the number of fusions between FFPE and paired FF materials (paired analysis *p*-value = 0.2, Figure 1A).

We also compared the number of uniquely mapped reads among the paired FF and FFPE sequenced libraries, a measure referred to as effective coverage for an RNA sequencing profile. On average, FFPE samples exhibited approximately twice the coverage of FF samples (*p*-value = 5.4 × 10^-^8^, Figure 1B). We did not observe a correlation between the number of fusion transcripts detected and the number of reads per library neither for FF, nor for FFPE samples (Figure 1C). Only fusions detected in at least two samples were included in this analysis.

Details on the numbers of uniquely mapped reads per sample and other mapping statistics are provided in Supplementary Table S2. Thus, the efficient detection of fusion transcripts in FFPE blocks, comparable to that in FF samples, may be at least partly due to the higher coverage by RNAseq reads. Interestingly, the median insert size was 20 bases shorter in FFPE than in FF samples, 186 vs. 206 bases, respectively (Supplementary Table S2, *p*-value = 3.9 × 10^-^7^), which could influence the fusion detection process due to the STAR-Fusion aligner properties.

Interestingly, perhaps due to the drastically different coverage, principal component analysis (PCA) revealed clearly separate clustering of the FF and FFPE gene expression profiles (Figure 1D). However, dendrogram analysis of pairwise distances primarily showed clustering that was specific to the sample IDs, rather than to the type of biomaterial, among the FF and FFPE biosamples (Supplementary Figure S1).

**FIGURE 2:**
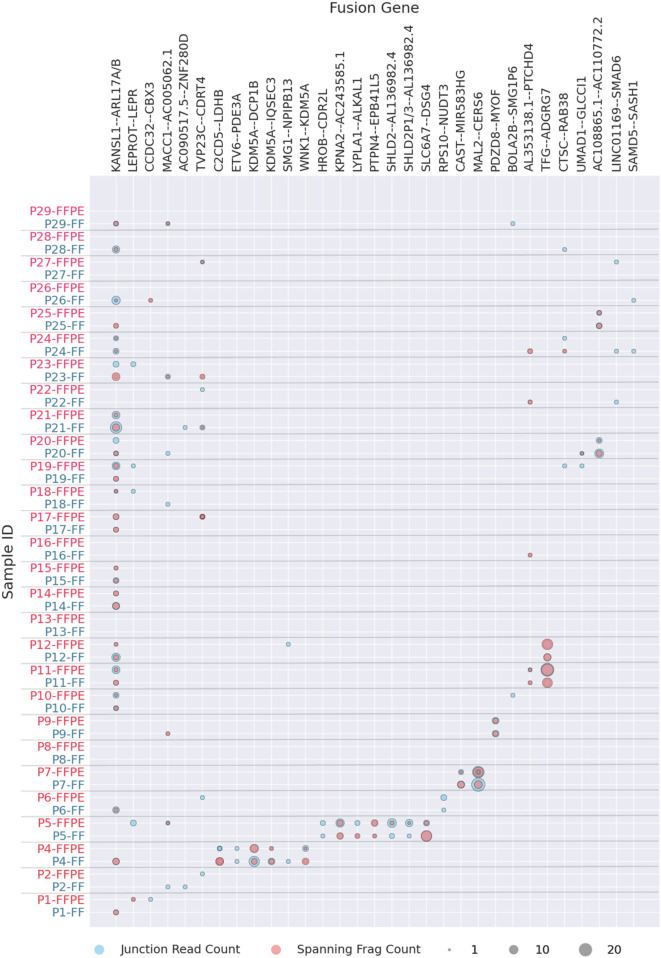
Fusion transcripts experimentally detected in FF or FFPE materials in more than one patient. Fusion statistics is ordered by patient ID, grey label showing FF and red label–FFPE biosamples.

The most commonly identified fusion transcripts in this study were *KANSL1-ARL17A/B* read-through transcripts, found in 20 patients (69%), followed by the fusions *MACC1-AC005062.1*, *LEPROT*-*LEPR*, *SMG1-NPIPB13*, and *AL353138.1-PTCHD4*, found in 7 (24%), 5 (17%), 3 (10%), and 3 (10%) patients, respectively (Figure 2; Supplementary Table S1). Of these, only the *KANSL1*-*ARL17A/B* fusion was previously reported in the literature (Zhou et al., 2017) while the others are newly identified or newly reported. Interestingly, *KANSL1-ARL17A/B* fusions have been detected not only in various solid and hematological cancers but also in patient-matched normal control tissues. Specifically, the *KANSL1-ARL17A* fusion has been associated with unfavorable outcomes in high-grade serous ovarian cancer (Newtson et al., 2021). Both the *KANSL1* and *ARL17A* genes are located on the reverse strand of chromosome 17 at the q21.31 locus. The frequent occurrence of *KANSL1-ARL17A/B* fusions may be attributed to two partial duplications of the *KANSL1* gene, which are prevalent at frequencies of 26% and 19%, respectively, in the European ancestry population (Boettger et al., 2012). This suggests that the mechanism of fusion generation could involve aberrant or alternative splicing of the two genes, rather than ongoing DNA rearrangement events (López-Nieva et al., 2019; Zhou et al., 2017).

The fusions that could be found in two patients were one known fusion *TFG-ADGRG7* and six new fusions *AC108865.1-AC110772.2*, *CCDC32-CBX3, CAST-AC104123.1, AC090517.5-ZNF280D, BOLA2B-SMG1P6,* and *UMAD1-GLCCI1* (Figure 2; Supplementary Table S1). Among these, the previously reported *TFG-ADGRG7* fusion could be also detected simultaneously in the normal and tumor samples (López-Nieva et al., 2019). Both fusion partners here are located on 3q12.2 genome locus.

Furthermore, except *CCDC32-CBX3* that most probably represented 15q15.1 – 7p15.2 translocation, all detected fusions occurring in at least two patient biosamples had fusion partners located in the same genomic region (Supplementary File S1). This strongly suggests read-through, duplication, or local deletion mechanisms for their generation. Many of them were presented by two or more alternative variants with different fusion sites (Supplementary File S1).

In total, of the 112 fusion transcripts detected in this study, 19 (17%) were previously documented in the ChimerSeq database of known fusions (Jang et al., 2020) or the Mitelman Database, while 93 (83%) were not previously reported in the literature or in the above repositories.

### Detection of the novel ALK fusion

**FIGURE 3:**
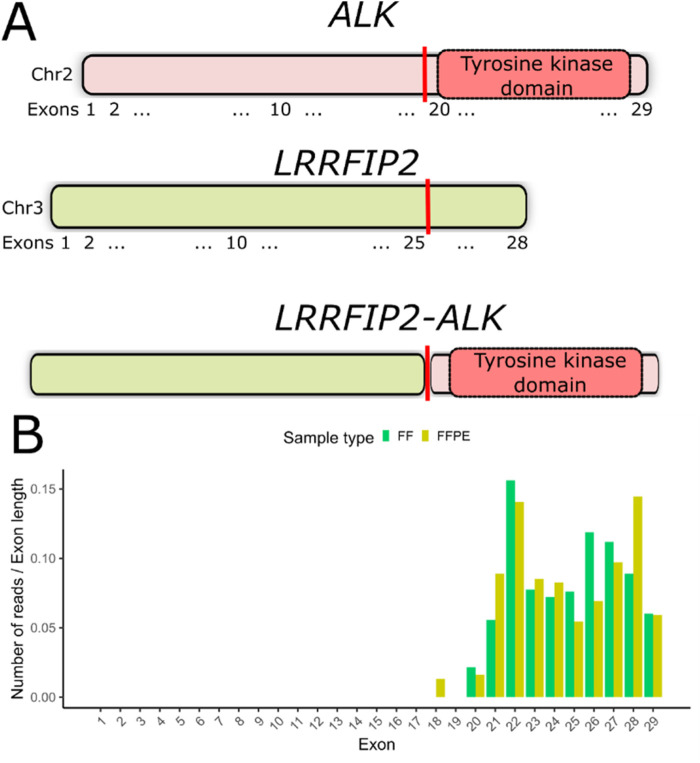
**(A)** Schematic representation of the *LRRFIP2-ALK* fusion transcript detected in patient P23. The vertical red line indicates the deduced fusion breakpoint. **(B)** Coverage of *ALK* gene exons by RNAseq reads in both FF and FFPE samples from patient #23. The number of counts mapped to exons has been normalized to exon lengths.

In the FF sample from patient P23, we detected an in-frame fusion transcript involving the *ALK* gene and *LRRFIP2*, which encodes the LRR-binding FLII-interacting protein 2. This fusion retains the entire tyrosine kinase domain spanning exons 20–29 of the *ALK* gene (Figure 3A, Supplementary File S1), suggesting the potential clinical efficacy of ALK inhibitors in this case. However, no supporting chimeric reads for this fusion were found in the FFPE sample of P23. We have previously demonstrated that an overall asymmetry in exon coverage by RNAseq reads of the 5′- and 3′-parts of a gene may indicate a gene fusion event (Rabushko et al., 2022). For patient P23, we observed a significant increase in *ALK* gene exon coverage beginning with exon 20 in both matched FF and FFPE RNAseq profiles (Figure 3B). We validated the presence of *LRRFIP2-ALK* fusion in both FF and FFPE samples using reverse transcription PCR followed by Sanger sequencing.

## Discussion

Detecting fusion events can be particularly challenging in FFPE tumor tissue samples. Fluorescent *in situ* hybridization (FISH) is commonly used to detect fusions in FFPE tissues, but this method is typically restricted to identifying known fusion pairs (Wagener-Ryczek and Pappesch, 2021). Alternative approaches, such as targeted RNA sequencing and various reverse transcription PCR-based methods, also face similar limitations in that they can only detect previously identified fusions (Wagener-Ryczek and Pappesch, 2021). Therefore, total RNA sequencing and/or whole-genome sequencing remain the only viable options for discovering novel gene fusions in both FFPE and FF tumor tissue materials (Yang et al., 2023).

We previously demonstrated that RNA sequencing of FFPE human tumor samples provides accurate gene expression profiling, establishing reproducible transcriptional patterns (Samii et al., 2021) and reliable quantification of cancer biomarkers (Sorokin et al., 2020a). However, to our knowledge, no published studies have directly compared the efficiency of fusion detection between FFPE and FF biomaterials. In our current study, we found that using FFPE materials resulted in a comparable number of fusion transcripts detected from total RNAseq data as with FF materials, although approximately twice as many reads were required for the FFPE libraries compared to the FF samples. Notably, the number of fusion transcripts identified in both FF and FFPE samples did not significantly differ. Interestingly, fusions identified in FF and FFPE samples from the same patient showed little overlap, suggesting that the STAR-Fusion software might not detect all existing fusions in the biosamples. The non-overlapping sets of chimeric transcripts could also be attributed to low expression levels of the transcripts and/or tumor heterogeneity.

Alternative RNA sequencing approaches that focus not only on detecting reads directly supporting a fusion event may significantly enhance the detection efficiency of chimeric cancer genes in FFPE samples. We have previously demonstrated that the pattern of exon coverage by RNAseq reads can be useful for identifying fusion genes, particularly when the major oncogenic partner (e.g., the gene moiety encoding the tyrosine kinase domain) is located on the 3′part of the chimera (Rabushko et al., 2022). In this study, we tested this approach and successfully identified a new, potentially clinically relevant *ALK* gene fusion in a matched FFPE sample as well.

*ALK*, a member of the insulin receptor superfamily of receptor tyrosine kinases, is composed of 29 exons, with exons 20–29 encoding the tyrosine kinase domain (Della Corte et al., 2018) *ALK* fusions are primarily found in lung cancer, where they occur with a prevalence of approximately 5% (Jazieh et al., 2021). The most frequent 5′fusion partner of *ALK* is *EML4*, which encodes the echinoderm microtubule-associated protein-like 4. Other common partners include *SQSTM1* (sequestosome), *DCTN1* (dynactin), *HIP1* (Huntington interacting protein 1), and *KIF5B* (kinesin family member 5B) (Shreenivas et al., 2023). Research indicates that the specific fusion partner may influence tumor sensitivity to ALK inhibitors (Childress et al., 2018). Although *ALK* fusions are less common in other tumor types, they have been occasionally detected in sarcomas, neuroblastoma, and esophageal, renal, breast, ovarian, thyroid, and colorectal cancers (Ross et al., 2017). In such cases, treatment with ALK-targeting drugs, such as crizotinib and alectinib, can lead to durable tumor responses (Childress et al., 2018).

In this study, we detected an *ALK* fusion with *LRRFIP2* as the 5′partner in a case of colorectal cancer. This same fusion was previously identified in one clinical case of epithelioid fibrous histiocytoma (Mansour et al., 2022). LRRFIP2, leucine-rich binding FLII interacting protein 2, is known to negatively regulate NLRP3 inflammasome activation in macrophages (Jin et al., 2013) and activate nuclear factor kappa B signaling by binding to the cytosolic tail of toll-like receptor 4 (Gunawardena et al., 2011). Notably, *LRRFIP2* has also been involved in fusions with *RAF1* in acral melanoma (LeBlanc et al., 2020) and with *MLH1* in hereditary non-polyposis colorectal cancer (Morak et al., 2011).

Using the bioinformatic tool STAR-Fusion (Haas et al., 2019), we identified the *LRRFIP2-ALK* fusion transcript in the FF sample but not in the FFPE sample of a patient. This discrepancy could be due to insufficient coverage, lower RNA integrity, tumor heterogeneity, or other factors. However, we detected a pattern of exon coverage by RNAseq reads that indicates the presence of this fusion in both FF and FFPE samples of this patient. Therefore, we conclude that inspecting exon coverage patterns for clinically relevant oncogenes can be valuable for characterizing FFPE-derived materials. This method can complement widely used software tools for detecting chimeric transcripts.

Since targeted therapies are available for less than a dozen oncogenic fusion types, such an inspection can even be performed manually when exon coverage is visualized. Additionally, an automated method for high-throughput exon coverage asymmetry analysis may be beneficial for batch detection of fusion gene candidates in FFPE RNAseq data. While this approach has limitations—it cannot identify the fusion partner or determine whether the open reading frame of a chimeric transcript is preserved—it can roughly identify the fusion breakpoint position and narrow the analysis to candidate cases requiring further in-depth investigation and molecular profiling.

## Data Availability

RNA sequencing profiles were deposited in NCBI Sequencing Read Archive (SRA) under accession ID PRJNA1208692.

## References

[B1] Boettger L. M. Handsaker R. E. Zody M. C. McCarroll S. A. (2012). Structural haplotypes and recent evolution of the human 17q21.31 region. Nat. Genet. 44, 881–885. 10.1038/ng.2334 22751096 PMC4020351

[B2] Bolstad B. M. (2017). preprocessCore: a collection of pre-processing functions. Available at: https://github.com/bmbolstad/preprocessCore (Accessed May 21, 2017).

[B3] Buzdin A. Sorokin M. Garazha A. Glusker A. Aleshin A. Poddubskaya E. (2020). RNA sequencing for research and diagnostics in clinical oncology. Semin. Cancer Biol. 60, 311–323. 10.1016/j.semcancer.2019.07.010 31412295

[B4] Cappello F. Angerilli V. Munari G. Ceccon C. Sabbadin M. Pagni F. (2022). FFPE-based NGS approaches into clinical practice: the limits of glory from a pathologist viewpoint. J. Pers. Med. 12, 750. 10.3390/jpm12050750 35629172 PMC9146170

[B5] Childress M. A. Himmelberg S. M. Chen H. Deng W. Davies M. A. Lovly C. M. (2018). ALK fusion partners impact response to ALK inhibition: differential effects on sensitivity, cellular phenotypes, and biochemical properties. Mol. Cancer Res. 16, 1724–1736. 10.1158/1541-7786.MCR-18-0171 30002191 PMC6214753

[B6] Della Corte C. M. Viscardi G. Di Liello R. Fasano M. Martinelli E. Troiani T. (2018). Role and targeting of anaplastic lymphoma kinase in cancer. Mol. Cancer 17, 30. 10.1186/s12943-018-0776-2 29455642 PMC5817803

[B7] Dobin A. Davis C. A. Schlesinger F. Drenkow J. Zaleski C. Jha S. (2013). STAR: ultrafast universal RNA-seq aligner. Bioinformatics 29, 15–21. 10.1093/bioinformatics/bts635 23104886 PMC3530905

[B8] Doebele R. C. Drilon A. Paz-Ares L. Siena S. Shaw A. T. Farago A. F. (2020). Entrectinib in patients with advanced or metastatic NTRK fusion-positive solid tumours: integrated analysis of three phase 1–2 trials. Lancet Oncol. 21, 271–282. 10.1016/S1470-2045(19)30691-6 31838007 PMC7461630

[B9] Dorney R. Dhungel B. P. Rasko J. E. J. Hebbard L. Schmitz U. (2023). Recent advances in cancer fusion transcript detection. Brief. Bioinform. 24, bbac519. 10.1093/bib/bbac519 36527429 PMC9851307

[B10] Drilon A. Laetsch T. W. Kummar S. DuBois S. G. Lassen U. N. Demetri G. D. (2018). Efficacy of larotrectinib in TRK fusion-positive cancers in adults and children. N. Engl. J. Med. 378, 731–739. 10.1056/NEJMoa1714448 29466156 PMC5857389

[B11] Gunawardena H. P. Huang Y. Kenjale R. Wang H. Xie L. Chen X. (2011). Unambiguous characterization of site-specific phosphorylation of leucine-rich repeat fli-I-interacting protein 2 (LRRFIP2) in toll-like receptor 4 (TLR4)-mediated signaling. J. Biol. Chem. 286, 10897–10910. 10.1074/jbc.M110.168179 21220426 PMC3064145

[B12] Haas B. J. Dobin A. Li B. Stransky N. Pochet N. Regev A. (2019). Accuracy assessment of fusion transcript detection via read-mapping and *de novo* fusion transcript assembly-based methods. Genome Biol. 20, 213. 10.1186/s13059-019-1842-9 31639029 PMC6802306

[B13] Hafstað V. Häkkinen J. Persson H. (2023). Fast and sensitive validation of fusion transcripts in whole-genome sequencing data. BMC Bioinforma. 24, 359. 10.1186/s12859-023-05489-5 PMC10518092 37741966

[B14] Haley L. Parimi V. Jiang L. Pallavajjala A. Hardy M. Yonescu R. (2021). Diagnostic utility of gene fusion panel to detect gene fusions in fresh and formalin-fixed, paraffin-embedded cancer specimens. J. Mol. Diagn. 23, 1343–1358. 10.1016/j.jmoldx.2021.07.015 34358677

[B15] Huang X. Li G. Li L. Wang J. Shen J. Chen Y. (2023). Establishing an RNA fusions panel in soft tissue sarcoma with clinical validation. Sci. Rep. 13, 4403. 10.1038/s41598-023-29511-1 36928336 PMC10020547

[B16] Jang Y. E. Jang I. Kim S. Cho S. Kim D. Kim K. (2020). ChimerDB 4.0: an updated and expanded database of fusion genes. Nucleic Acids Res. 48, D817–D824. 10.1093/nar/gkz1013 31680157 PMC7145594

[B17] Javle M. Roychowdhury S. Kelley R. K. Sadeghi S. Macarulla T. Weiss K. H. (2021). Infigratinib (BGJ398) in previously treated patients with advanced or metastatic cholangiocarcinoma with FGFR2 fusions or rearrangements: mature results from a multicentre, open-label, single-arm, phase 2 study. Lancet Gastroenterol. Hepatol. 6, 803–815. 10.1016/S2468-1253(21)00196-5 34358484

[B18] Jazieh A. R. Gaafar R. Errihani H. Jaafar H. Al Dayel F. Bahnassy A. A. (2021). Real-world data on the prevalence of anaplastic lymphoma kinase–positive non–small-cell lung cancer in the Middle East and north africa. JCO Glob. Oncol. 7, 1556–1563. 10.1200/GO.21.00067 34788123 PMC8613346

[B19] Jin J. Yu Q. Han C. Hu X. Xu S. Wang Q. (2013). LRRFIP2 negatively regulates NLRP3 inflammasome activation in macrophages by promoting Flightless-I-mediated caspase-1 inhibition. Nat. Commun. 4, 2075. 10.1038/ncomms3075 23942110 PMC3753543

[B20] Lazar V. Zhang B. Magidi S. Le Tourneau C. Raymond E. Ducreux M. (2023). A transcriptomics approach to expand therapeutic options and optimize clinical trials in oncology. Ther. Adv. Med. Oncol. 15, 17588359231156382. 10.1177/17588359231156382 37025260 PMC10071163

[B21] LeBlanc R. E. Lefferts J. A. Baker M. L. Linos K. D. (2020). Novel LRRFIP2-RAF1 fusion identified in an acral melanoma: a review of the literature on melanocytic proliferations with RAF1 fusions and the potential therapeutic implications. J. Cutan. Pathol. 47, 1181–1186. 10.1111/cup.13817 32700768

[B22] Li J. Lu H. Ng P. K.-S. Pantazi A. Ip C. K. M. Jeong K. J. (2023). A functional genomic approach to actionable gene fusions for precision oncology. Sci. Adv. 8, eabm2382. 10.1126/sciadv.abm2382 PMC8827659 35138907

[B23] López-Nieva P. Fernández-Navarro P. Graña-Castro O. Andrés-León E. Santos J. Villa-Morales M. (2019). Detection of novel fusion-transcripts by RNA-Seq in T-cell lymphoblastic lymphoma. Sci. Rep. 9, 5179. 10.1038/s41598-019-41675-3 30914738 PMC6435891

[B24] Loriot Y. Necchi A. Park S. H. Garcia-Donas J. Huddart R. Burgess E. (2019). Erdafitinib in locally advanced or metastatic urothelial carcinoma. N. Engl. J. Med. 381, 338–348. 10.1056/NEJMoa1817323 31340094

[B25] Mansour B. Donati M. Michalová K. Michal M. Ptáková N. Hájková V. (2022). Epithelioid fibrous histiocytoma: three diagnostically challenging cases with novel ALK gene fusions, unusual storiform growth pattern, and a prominent spindled morphology. Virchows Arch. 481, 751–757. 10.1007/s00428-022-03418-0 36171493

[B26] Morak M. Massdorf T. Locher M. Holinski-Feder E. (2011). Disease-causing gene-flanking genomic rearrangements in HNPCC patients. Hered. Cancer Clin. Pract. 9, P28. 10.1186/1897-4287-9-S1-P28

[B27] Newtson A. Reyes H. Devor E. J. Goodheart M. J. Bosquet J. G. (2021). Identification of novel fusion transcripts in high grade serous ovarian cancer. Int. J. Mol. Sci. 22, 4791. 10.3390/ijms22094791 33946483 PMC8125626

[B28] Rabushko E. Sorokin M. Suntsova M. Seryakov A. P. Kuzmin D. V. Poddubskaya E. (2022). Experimentally deduced criteria for detection of clinically relevant fusion 3′ oncogenes from FFPE bulk RNA sequencing data. Biomedicines 10, 1866. 10.3390/biomedicines10081866 36009413 PMC9405289

[B29] Ross J. S. Ali S. M. Fasan O. Block J. Pal S. Elvin J. A. (2017). ALK fusions in a wide variety of tumor types respond to anti-ALK targeted therapy. Oncologist 22, 1444–1450. 10.1634/theoncologist.2016-0488 29079636 PMC5728036

[B30] Samii A. Sorokin M. Kar S. Makovskaia L. Garazha A. Hartmann C. (2021). Case of multifocal glioblastoma with four fusion transcripts of ALK, FGFR2, NTRK2, and NTRK3 genes stresses the need for tumor tissue multisampling for transcriptomic analysis. Cold Spring Harb. Mol. case Stud. 7, a006100. 10.1101/MCS.A006100 34341009 PMC8327882

[B31] Schubert L. Elliott A. Le A. T. Estrada-Bernal A. Doebele R. C. Lou E. (2023). ERBB family fusions are recurrent and actionable oncogenic targets across cancer types. Front. Oncol. 13, 1115405. 10.3389/fonc.2023.1115405 37168365 PMC10164992

[B32] Shaw A. T. Ou S.-H. I. Bang Y.-J. Camidge D. R. Solomon B. J. Salgia R. (2014). Crizotinib in ROS1-rearranged non-small-cell lung cancer. N. Engl. J. Med. 371, 1963–1971. 10.1056/NEJMoa1406766 25264305 PMC4264527

[B33] Shreenivas A. Janku F. Gouda M. A. Chen H.-Z. George B. Kato S. (2023). ALK fusions in the pan-cancer setting: another tumor-agnostic target? Precis. Oncol. 7, 101. 10.1038/s41698-023-00449-x PMC10542332 37773318

[B34] Sorokin M. Gorelyshev A. Efimov V. Zotova E. Zolotovskaia M. Rabushko E. (2021). RNA sequencing data for FFPE tumor blocks can Be used for robust estimation of tumor mutation burden in individual biosamples. Front. Oncol. 11, 732644. 10.3389/fonc.2021.732644 34650919 PMC8506044

[B35] Sorokin M. Ignatev K. Poddubskaya E. Vladimirova U. Gaifullin N. Lantsov D. (2020a). RNA sequencing in comparison to immunohistochemistry for measuring cancer biomarkers in breast cancer and lung cancer specimens. Biomedicines 8, 114. 10.3390/BIOMEDICINES8050114 32397474 PMC7277916

[B36] Sorokin M. Poddubskaya E. Baranova M. Glusker A. Kogoniya L. Markarova E. (2020b). RNA sequencing profiles and diagnostic signatures linked with response to ramucirumab in gastric cancer. Mol. Case Stud. 6, a004945. mcs.a004945. 10.1101/mcs.a004945 PMC7133748 32060041

[B37] Sorokin M. Rabushko E. Rozenberg J. M. Mohammad T. Seryakov A. Sekacheva M. (2022). Clinically relevant fusion oncogenes: detection and practical implications. Ther. Adv. Med. Oncol. 14, 17588359221144108. 10.1177/17588359221144108 36601633 PMC9806411

[B38] Subbiah V. Cassier P. A. Siena S. Garralda E. Paz-Ares L. Garrido P. (2022a). Pan-cancer efficacy of pralsetinib in patients with RET fusion–positive solid tumors from the phase 1/2 ARROW trial. Nat. Med. 28, 1640–1645. 10.1038/s41591-022-01931-y 35962206 PMC9388374

[B39] Subbiah V. Wolf J. Konda B. Kang H. Spira A. Weiss J. (2022b). Tumour-agnostic efficacy and safety of selpercatinib in patients with RET fusion-positive solid tumours other than lung or thyroid tumours (LIBRETTO-001): a phase 1/2, open-label, basket trial. Lancet Oncol. 23, 1261–1273. 10.1016/S1470-2045(22)00541-1 36108661 PMC11702314

[B40] Suntsova M. Gaifullin N. Allina D. Reshetun A. Li X. Mendeleeva L. (2019). Atlas of RNA sequencing profiles for normal human tissues. Sci. data 6, 36. 10.1038/s41597-019-0043-4 31015567 PMC6478850

[B41] Wagener-Ryczek S. Pappesch R. (2021). Targeted RNA-sequencing for the evaluation of gene fusions in lung tumors: current status and future prospects. Expert Rev. Mol. diagn. 21, 531–534. 10.1080/14737159.2021.1920399 33887162

[B42] Walden D. Eslinger C. Bekaii-Saab T. (2022). Pemigatinib for adults with previously treated, locally advanced or metastatic cholangiocarcinoma with FGFR2 fusions/rearrangements. Ther. Adv. Gastroenterol. 15, 17562848221115317. 10.1177/17562848221115317 PMC9364186 35967919

[B43] Wu J. Savooji J. Liu D. (2016). Second- and third-generation ALK inhibitors for non-small cell lung cancer. J. Hematol. Oncol. 9, 19. 10.1186/s13045-016-0251-8 26951079 PMC4782349

[B44] Yang Y. Shu Y. Tang Y. Zhao S. Jia Y. Ji J. (2023). RNA sequencing of myeloid sarcoma, shed light on myeloid sarcoma stratification. Cancer Med. 12, 9156–9166. 10.1002/cam4.5654 36916780 PMC10166975

[B45] Zhou J. X. Yang X. Ning S. Wang L. Wang K. Zhang Y. (2017). Identification of KANSARL as the first cancer predisposition fusion gene specific to the population of European ancestry origin. Oncotarget 8 (31), 50594–50607. 10.18632/oncotarget.16385 28881586 PMC5584173

[B46] Zhu G. Benayed R. Ho C. Mullaney K. Sukhadia P. Rios K. (2019). Diagnosis of known sarcoma fusions and novel fusion partners by targeted RNA sequencing with identification of a recurrent ACTB-FOSB fusion in pseudomyogenic hemangioendothelioma. Mod. Pathol. 32, 609–620. 10.1038/s41379-018-0175-7 30459475 PMC6486453

