# Supplementary material for: Detection of fusion events by RNA sequencing in FFPE versus freshly frozen colorectal cancer tissue samples
Source: Front Mol Biosci. 2025 Jan 21;11:1448792. doi: 10.3389/fmolb.2024.1448792 (PMC11791353; doi:10.3389/fmolb.2024.1448792)
Supplement: Supplementary file 2 [file DataSheet1.pdf]

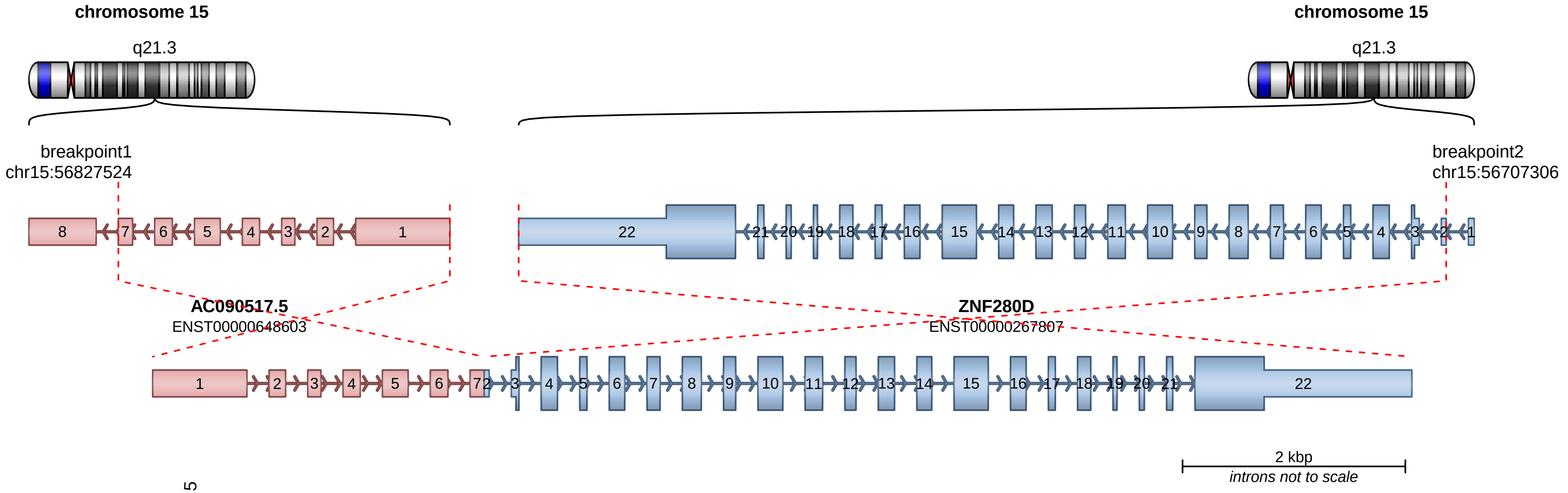

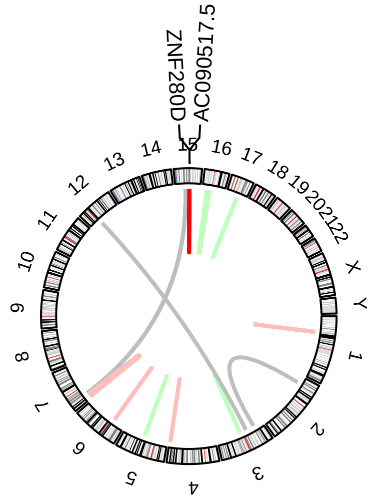

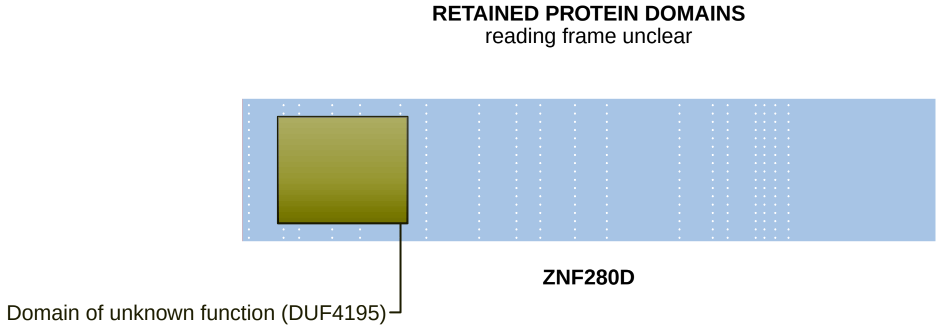

SUPPORTING READ COUNT

Split reads = 1
Discordant mates = 0

translocation
duplication
deletion
inversion

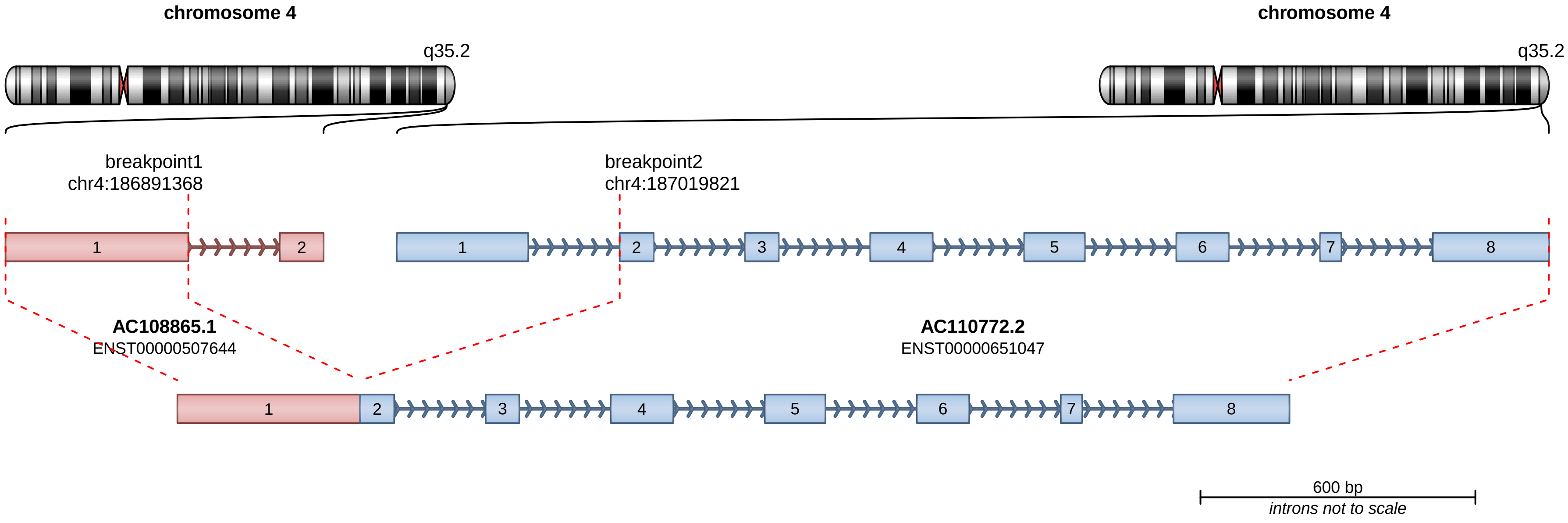

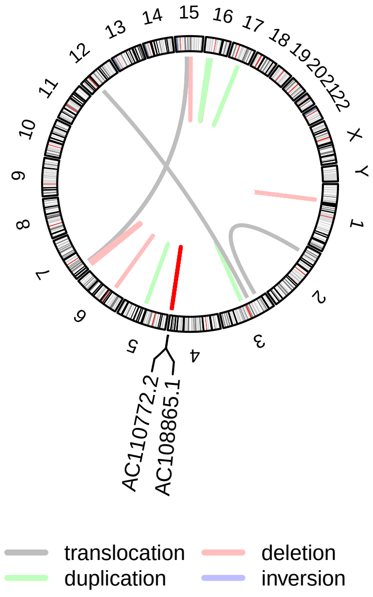

Genes are not protein−coding.

SUPPORTING READ COUNT

Split reads = 1
Discordant mates = 0

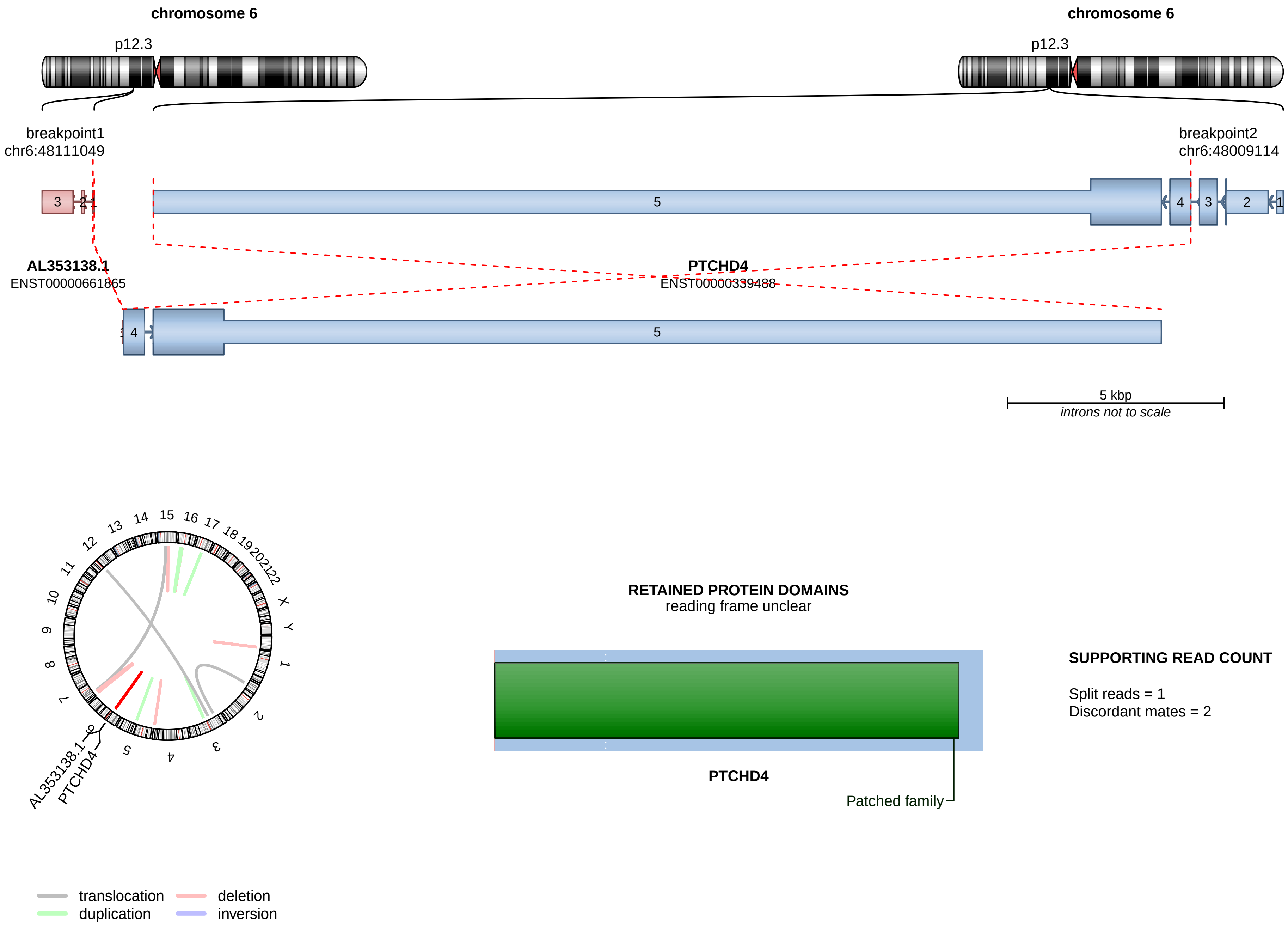

chromosome 6
p12.3
chromosome 6
p12.3
breakpoint1
chr6:48111049
breakpoint2
chr6:48009114
AL353138.1
ENST00000661865
PTCHD4
ENST00000339488
5 kbp
introns not to scale
AL353138.1
PTCHD4
RETAINED PROTEIN DOMAINS
reading frame unclear
PTCHD4
Patched family
SUPPORTING READ COUNT
Split reads = 1
Discordant mates = 2
translocation
duplication
deletion
inversion

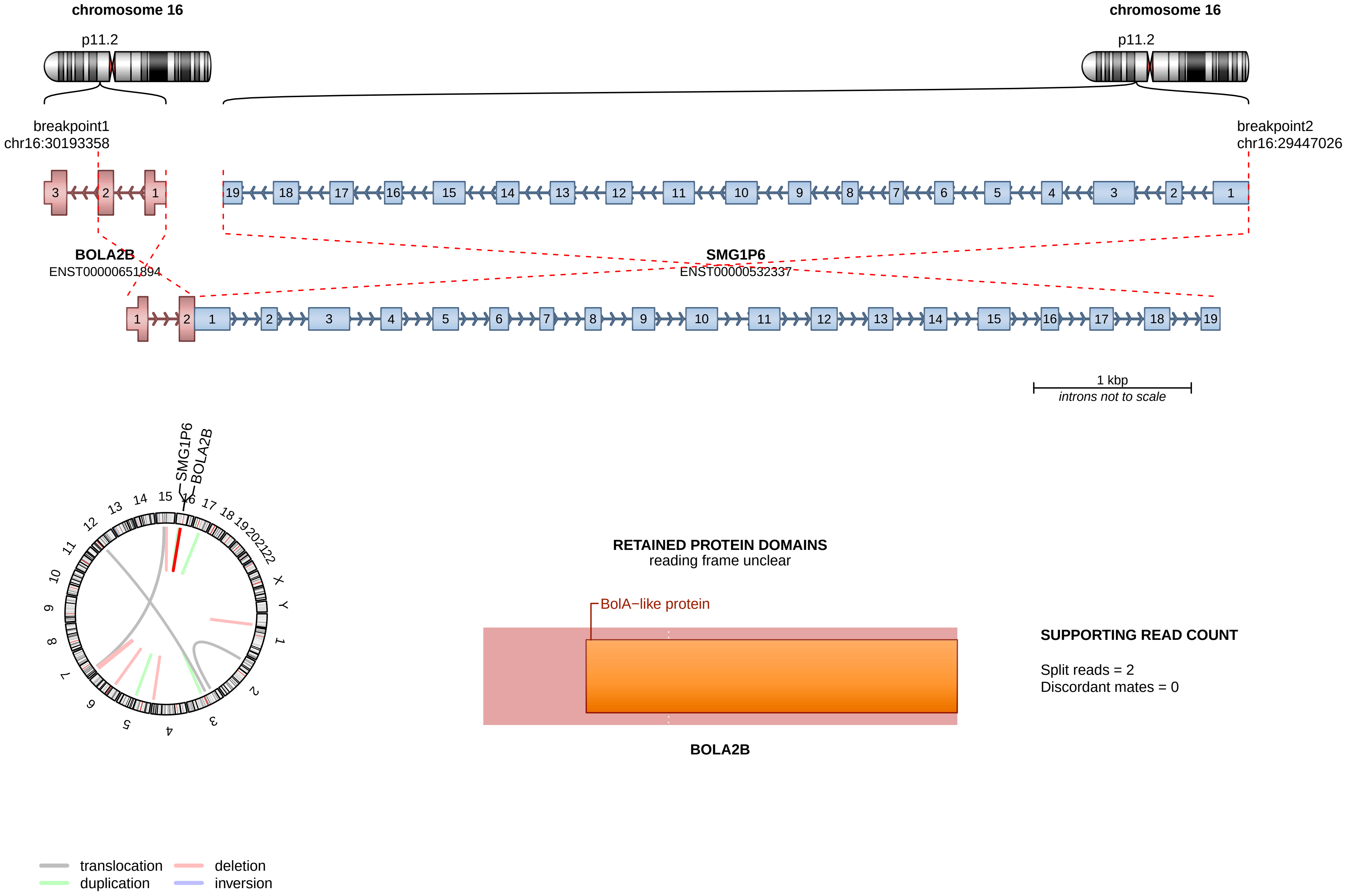
chromosome 16
p11.2
chromosome 16
p11.2
breakpoint1
chr16:30193358
breakpoint2
chr16:29447026
BOLA2B
ENST00000651894
SMG1P6
ENST00000532337
1 kbp
introns not to scale
SMG1P6
BOLA2B
RETAINED PROTEIN DOMAINS
reading frame unclear
BolA−like protein
BOLA2B
SUPPORTING READ COUNT
Split reads = 2
Discordant mates = 0
translocation
duplication
deletion
inversion

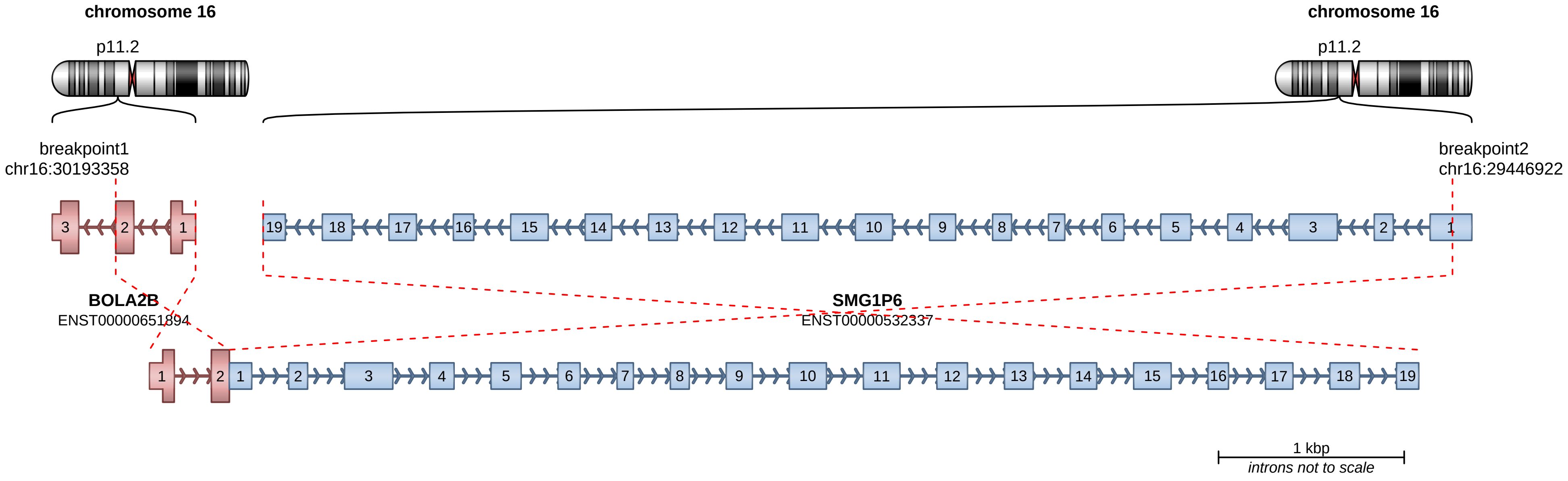

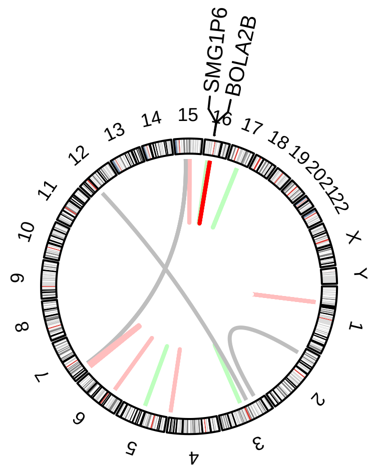

translocation
duplication
deletion
inversion

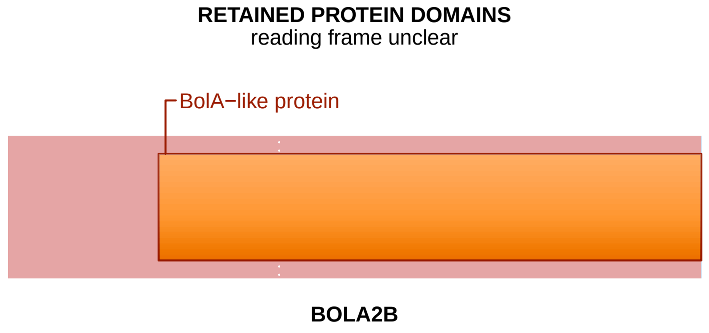

SUPPORTING READ COUNT

Split reads = 2
Discordant mates = 0

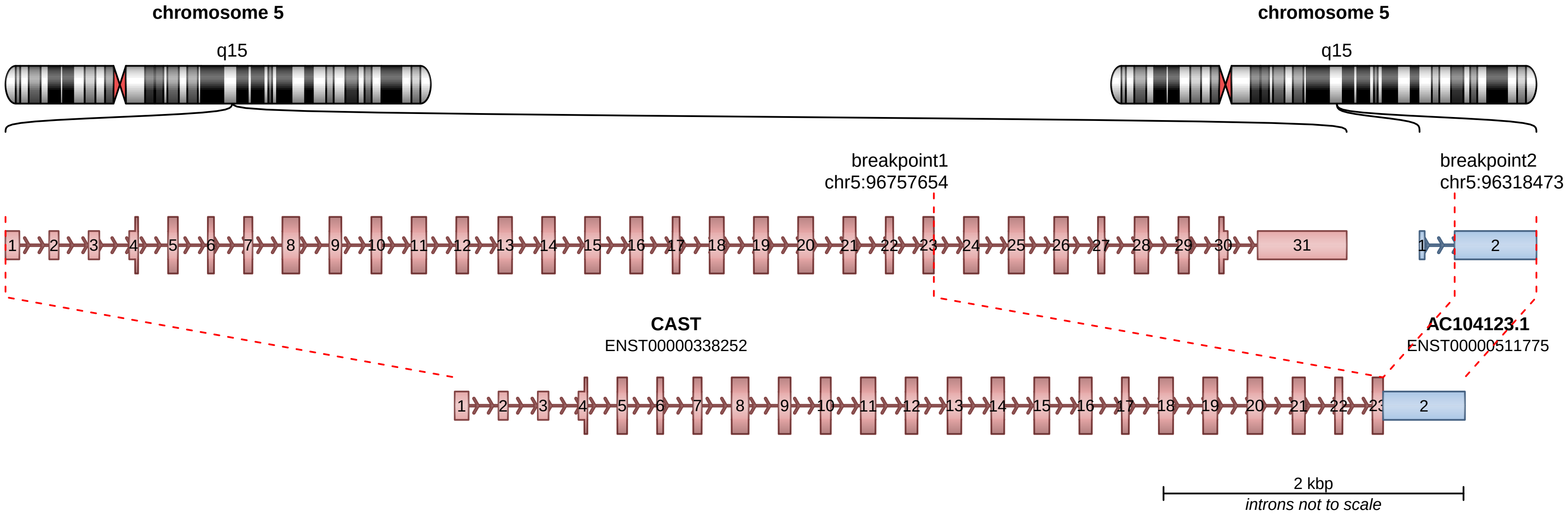

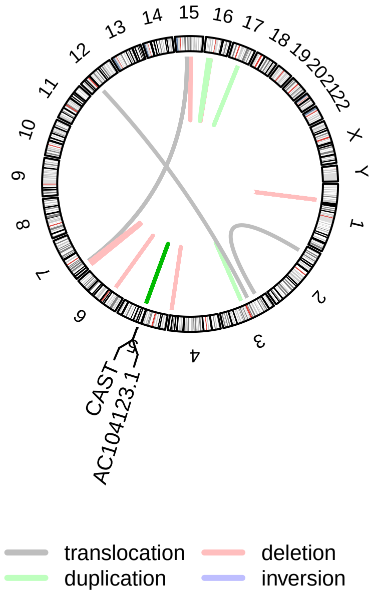

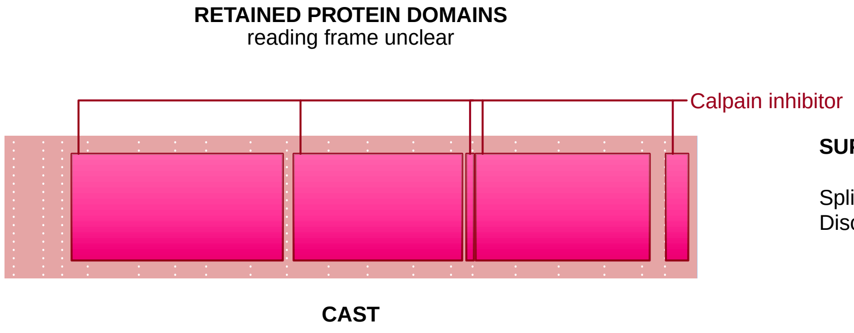

SUPPORTING READ COUNT

Split reads = 2
Discordant mates = 0

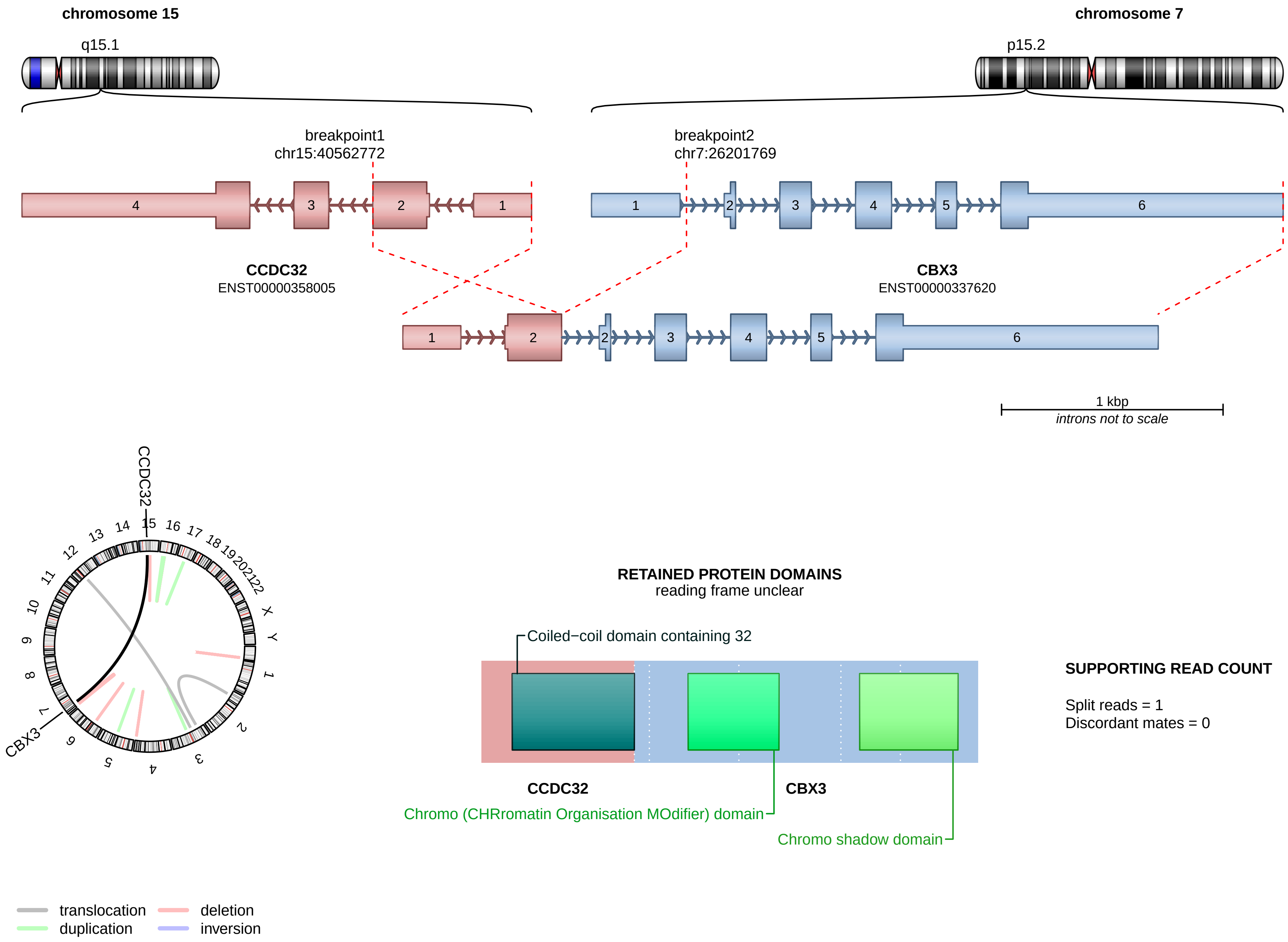

chromosome 15
q15.1
chromosome 7
p15.2
breakpoint1
chr15:40562772
breakpoint2
chr7:26201769
4
3
2
1
1
2
3
4
5
6
CCDC32
ENST00000358005
CBX3
ENST00000337620
1
2
2
3
4
5
6
1 kbp
introns not to scale
CCDC32
CBX3
1 2 3 4 5 6 7 8 9 10 11 12 13 14 15 16 17 18 19 20 21 22 X Y
translocation
duplication
deletion
inversion
RETAINED PROTEIN DOMAINS
reading frame unclear
Coiled−coil domain containing 32
CCDC32
CBX3
Chromo (CHRromatin Organisation MOdifier) domain
Chromo shadow domain
SUPPORTING READ COUNT
Split reads = 1
Discordant mates = 0

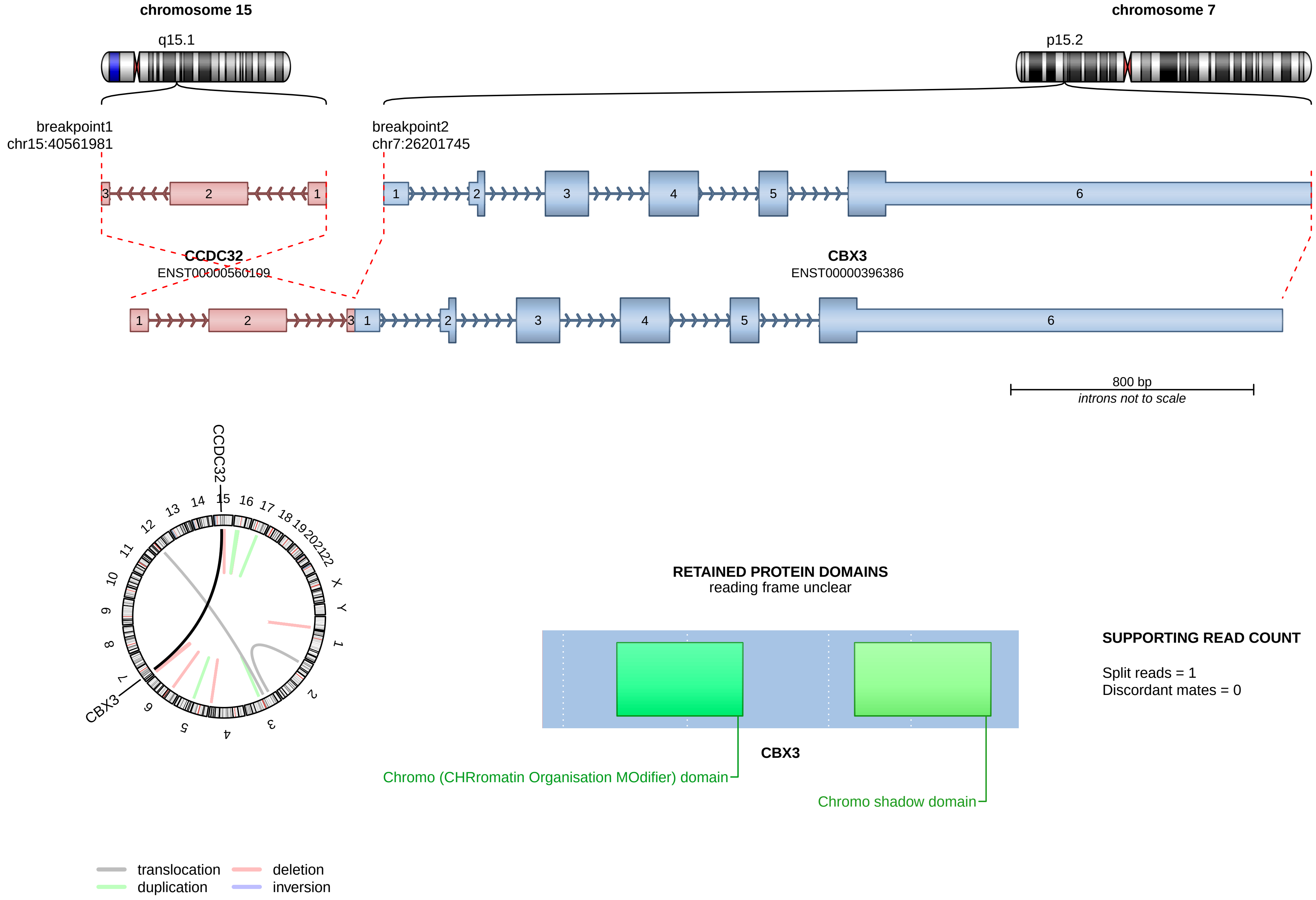
chromosome 15
q15.1
chromosome 7
p15.2
breakpoint1
chr15:40561981
breakpoint2
chr7:26201745
CCDC32
ENST00000560109
CBX3
ENST00000396386
800 bp
introns not to scale
CCDC32
CBX3
RETAINED PROTEIN DOMAINS
reading frame unclear
CBX3
Chromo (CHRromatin Organisation MOdifier) domain
Chromo shadow domain
SUPPORTING READ COUNT
Split reads = 1
Discordant mates = 0
translocation
deletion
duplication
inversion

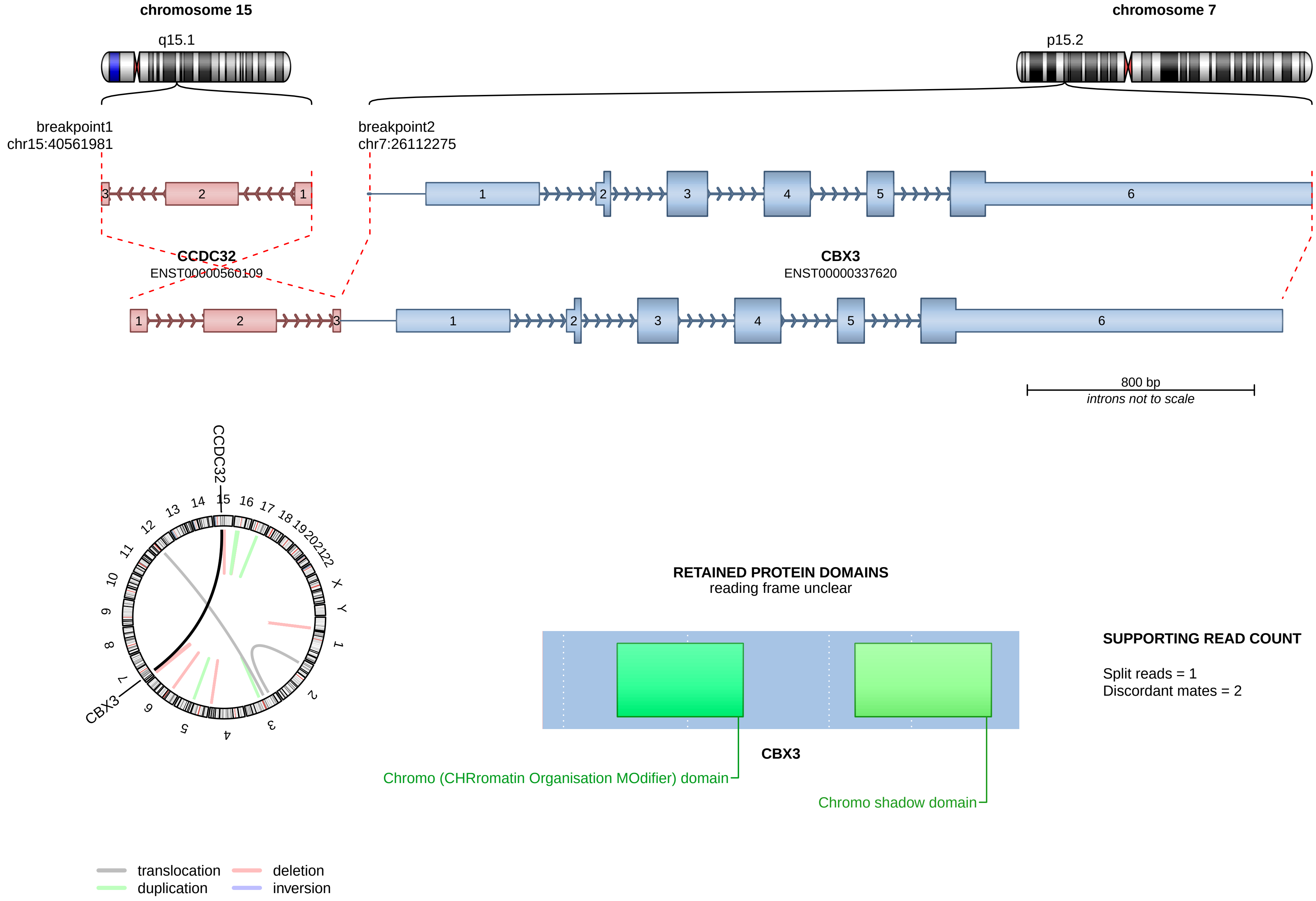
chromosome 15
q15.1
chromosome 7
p15.2
breakpoint1
chr15:40561981
breakpoint2
chr7:26112275
CCDC32
ENST00000560109
CBX3
ENST00000337620
800 bp
introns not to scale
CCDC32
CBX3
translocation
duplication
deletion
inversion
RETAINED PROTEIN DOMAINS
reading frame unclear
CBX3
Chromo (CHRromatin Organisation MOdifier) domain
Chromo shadow domain
SUPPORTING READ COUNT
Split reads = 1
Discordant mates = 2

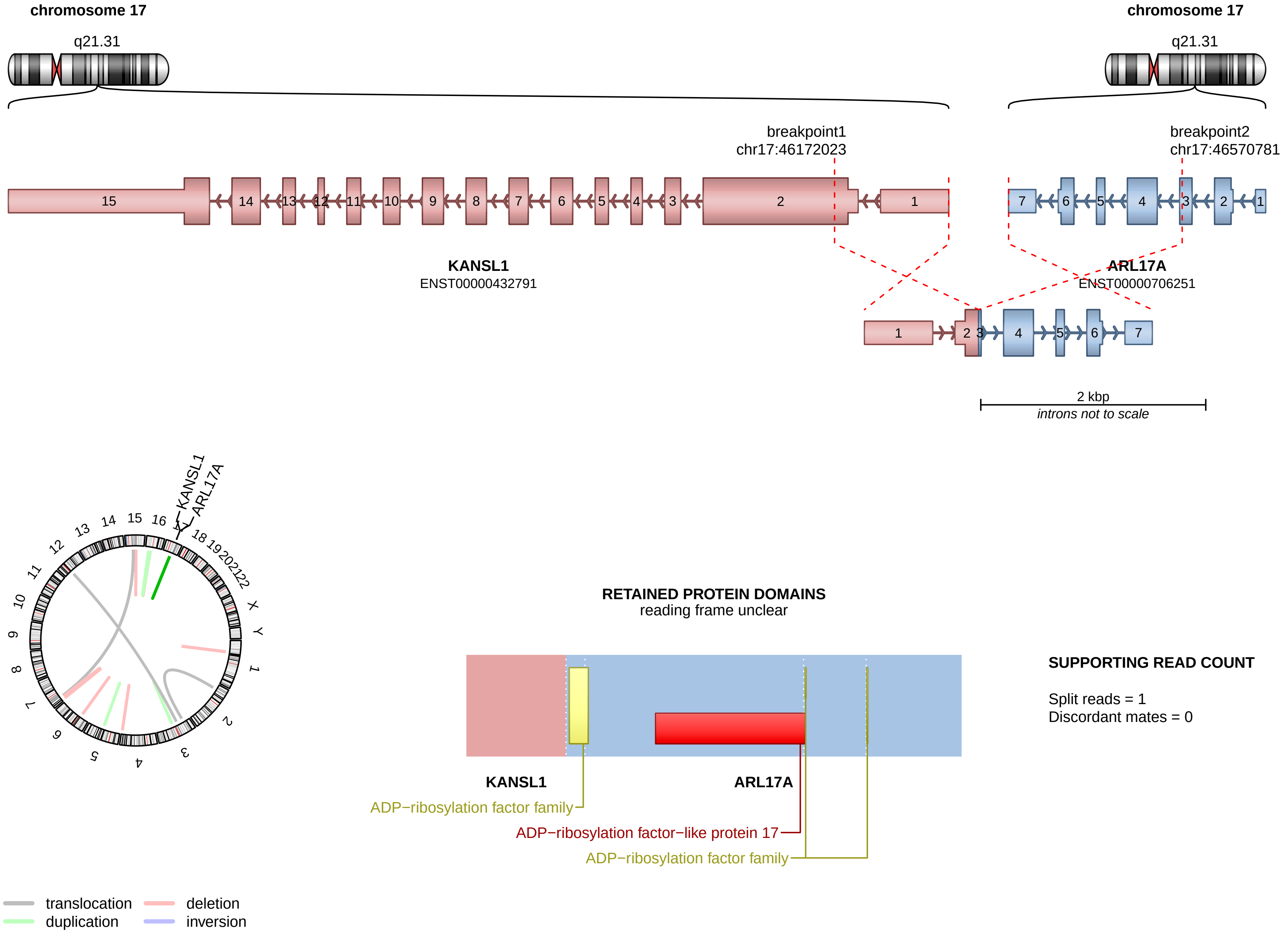
chromosome 17
q21.31
chromosome 17
q21.31
breakpoint1
chr17:46172023
breakpoint2
chr17:46570781
KANSL1
ENST00000432791
ARL17A
ENST00000706251
2 kbp
introns not to scale
KANSL1
ARL17A
RETAINED PROTEIN DOMAINS
reading frame unclear
KANSL1
ARL17A
ADP−ribosylation factor family
ADP−ribosylation factor−like protein 17
ADP−ribosylation factor family
SUPPORTING READ COUNT
Split reads = 1
Discordant mates = 0
translocation
deletion
duplication
inversion

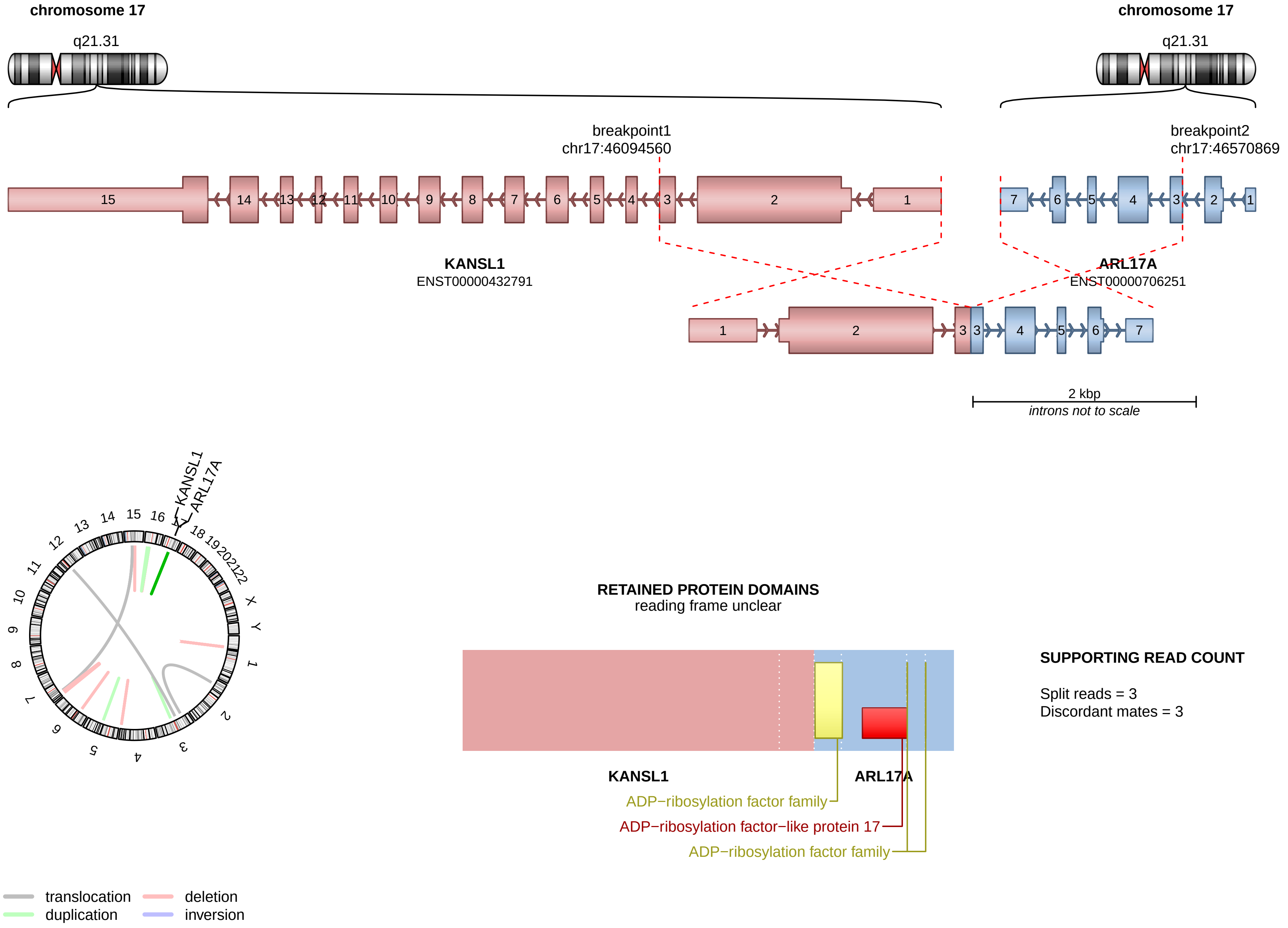
chromosome 17
q21.31
chromosome 17
q21.31
breakpoint1
chr17:46094560
breakpoint2
chr17:46570869
15
14
13
12
11
10
9
8
7
6
5
4
3
2
1
7
6
5
4
3
2
1
KANSL1
ENST00000432791
ARL17A
ENST00000706251
1
2
3
3
4
5
6
7
2 kbp
introns not to scale
KANSL1
ARL17A
RETAINED PROTEIN DOMAINS
reading frame unclear
KANSL1
ARL17A
ADP−ribosylation factor family
ADP−ribosylation factor−like protein 17
ADP−ribosylation factor family
SUPPORTING READ COUNT
Split reads = 3
Discordant mates = 3
translocation
duplication
deletion
inversion

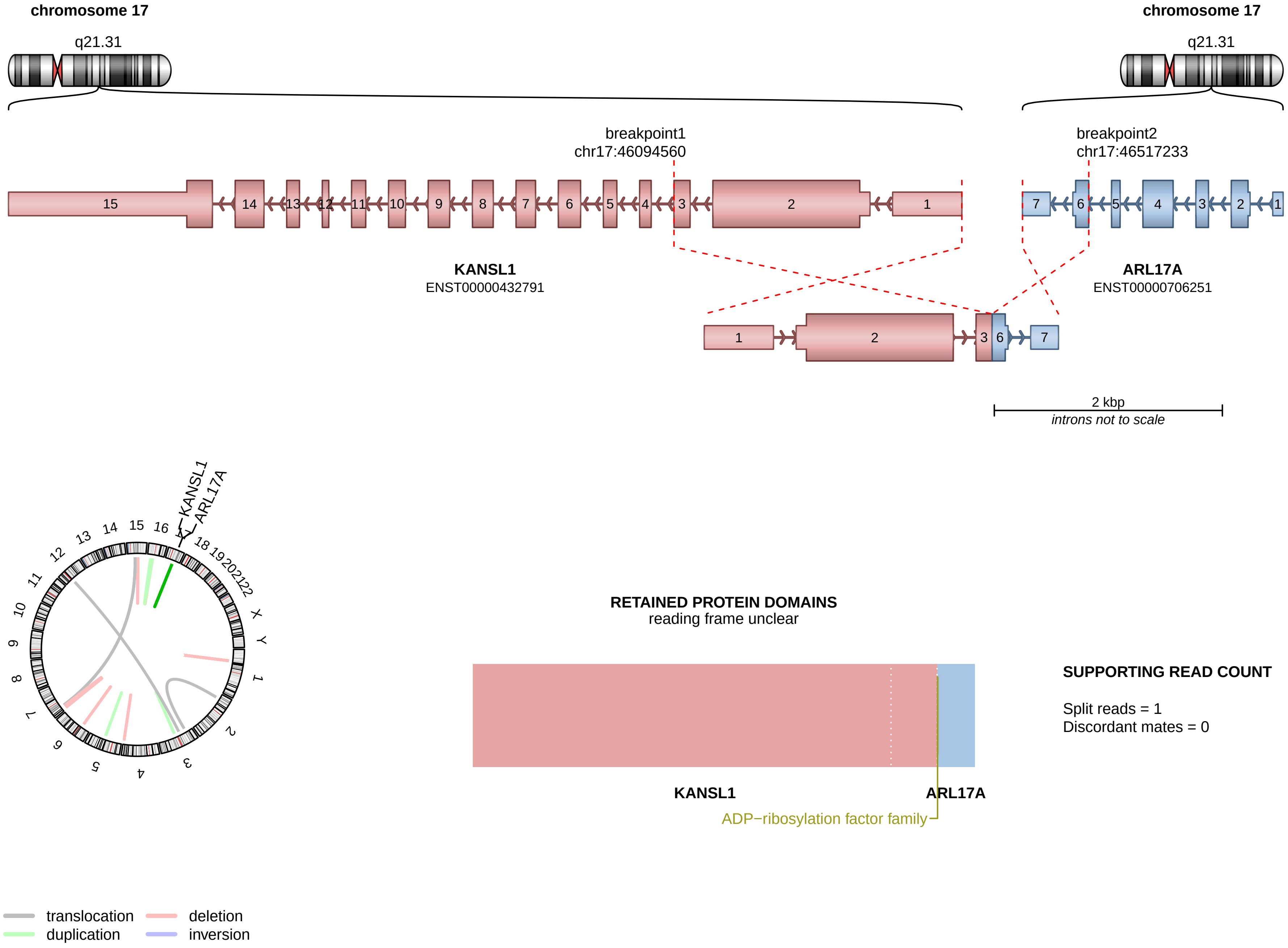
chromosome 17
q21.31
chromosome 17
q21.31
breakpoint1
chr17:46094560
breakpoint2
chr17:46517233
15
14
13
12
11
10
9
8
7
6
5
4
3
2
1
7
6
5
4
3
2
1
KANSL1
ENST00000432791
ARL17A
ENST00000706251
1
2
3
6
7
2 kbp
introns not to scale
KANSL1
ARL17A
RETAINED PROTEIN DOMAINS
reading frame unclear
SUPPORTING READ COUNT
Split reads = 1
Discordant mates = 0
KANSL1
ARL17A
ADP−ribosylation factor family
translocation
deletion
duplication
inversion

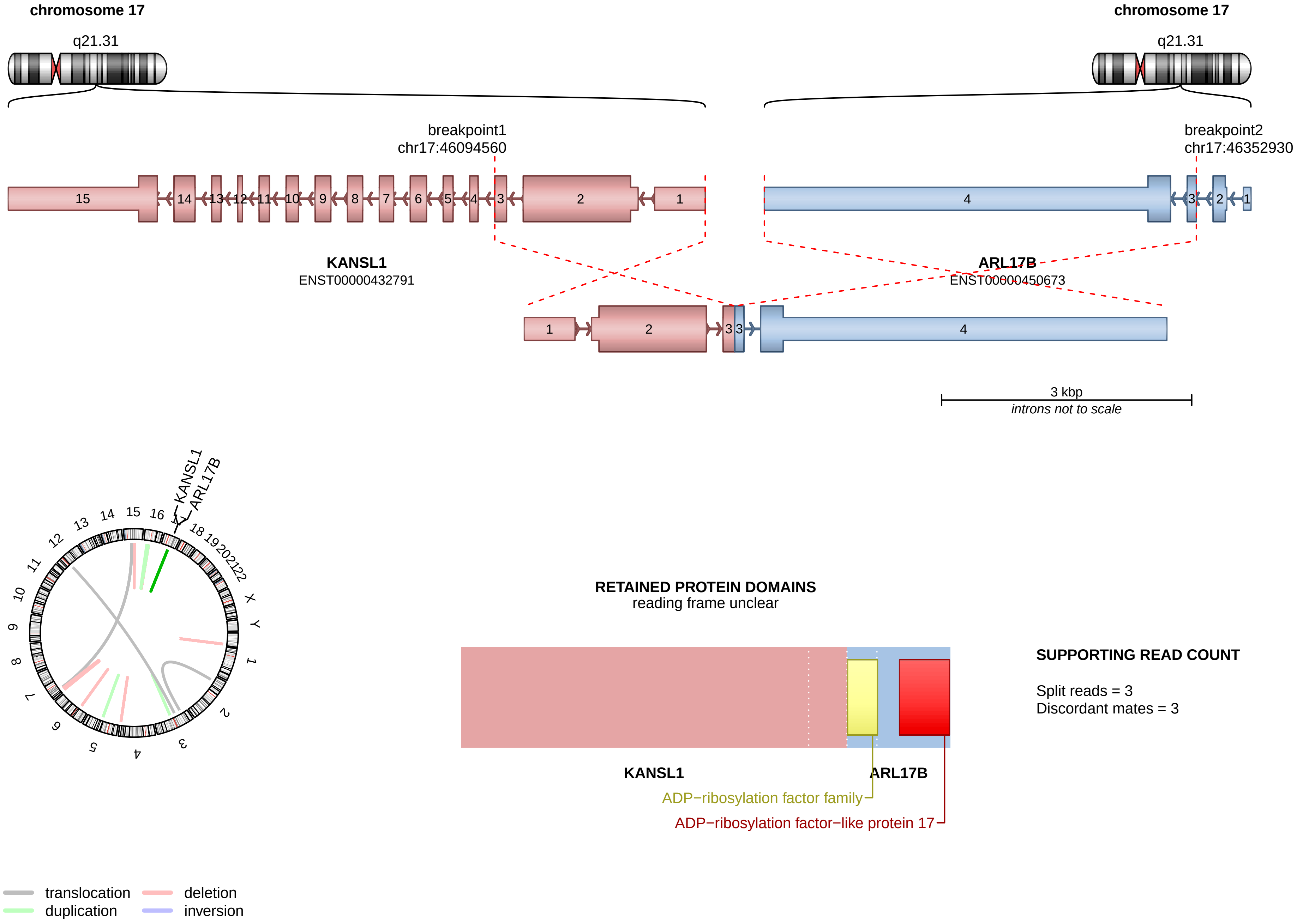
chromosome 17
q21.31
chromosome 17
q21.31
breakpoint1
chr17:46094560
breakpoint2
chr17:46352930
15
14
13
12
11
10
9
8
7
6
5
4
3
2
1
4
3
2
1
KANSL1
ENST00000432791
ARL17B
ENST00000450673
1
2
3
3
4
3 kbp
introns not to scale
KANSL1
ARL17B
RETAINED PROTEIN DOMAINS
reading frame unclear
KANSL1
ARL17B
ADP−ribosylation factor family
ADP−ribosylation factor−like protein 17
SUPPORTING READ COUNT
Split reads = 3
Discordant mates = 3
translocation
deletion
duplication
inversion

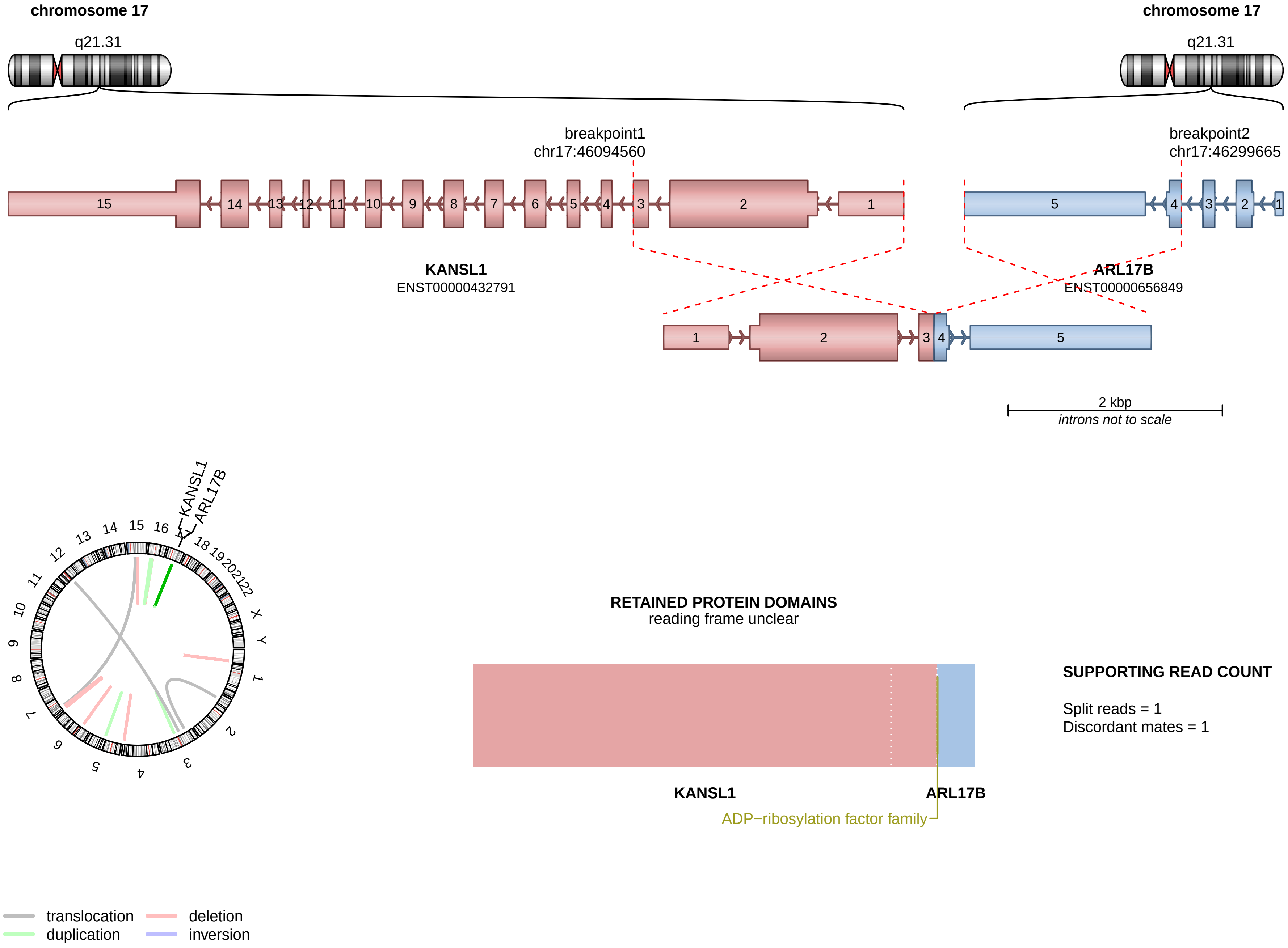
chromosome 17
q21.31
chromosome 17
q21.31
breakpoint1
chr17:46094560
breakpoint2
chr17:46299665
15
14
13
12
11
10
9
8
7
6
5
4
3
2
1
5
4
3
2
1
KANSL1
ENST00000432791
ARL17B
ENST00000656849
1
2
3
4
5
2 kbp
introns not to scale
KANSL1
ARL17B
1
2
3
4
5
6
7
8
9
10
11
12
13
14
15
16
17
18
19
20
21
22
X
Y
RETAINED PROTEIN DOMAINS
reading frame unclear
KANSL1
ARL17B
ADP−ribosylation factor family
SUPPORTING READ COUNT
Split reads = 1
Discordant mates = 1
translocation
duplication
deletion
inversion

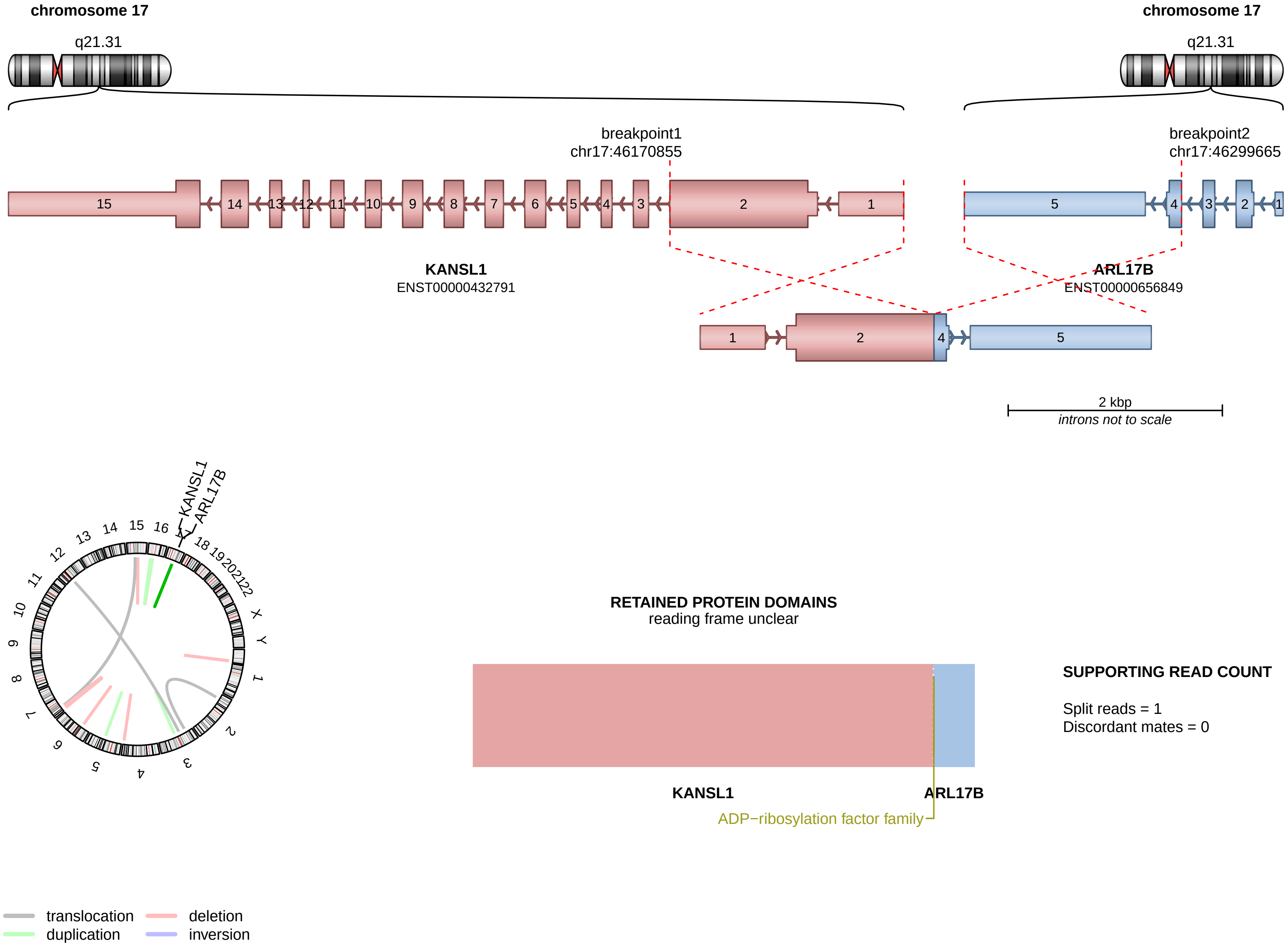
chromosome 17
q21.31
chromosome 17
q21.31
breakpoint1
chr17:46170855
breakpoint2
chr17:46299665
15
14
13
12
11
10
9
8
7
6
5
4
3
2
1
5
4
3
2
1
KANSL1
ENST00000432791
ARL17B
ENST00000656849
1
2
4
5
2 kbp
introns not to scale
KANSL1
ARL17B
RETAINED PROTEIN DOMAINS
reading frame unclear
SUPPORTING READ COUNT
Split reads = 1
Discordant mates = 0
KANSL1
ARL17B
ADP−ribosylation factor family
translocation
deletion
duplication
inversion

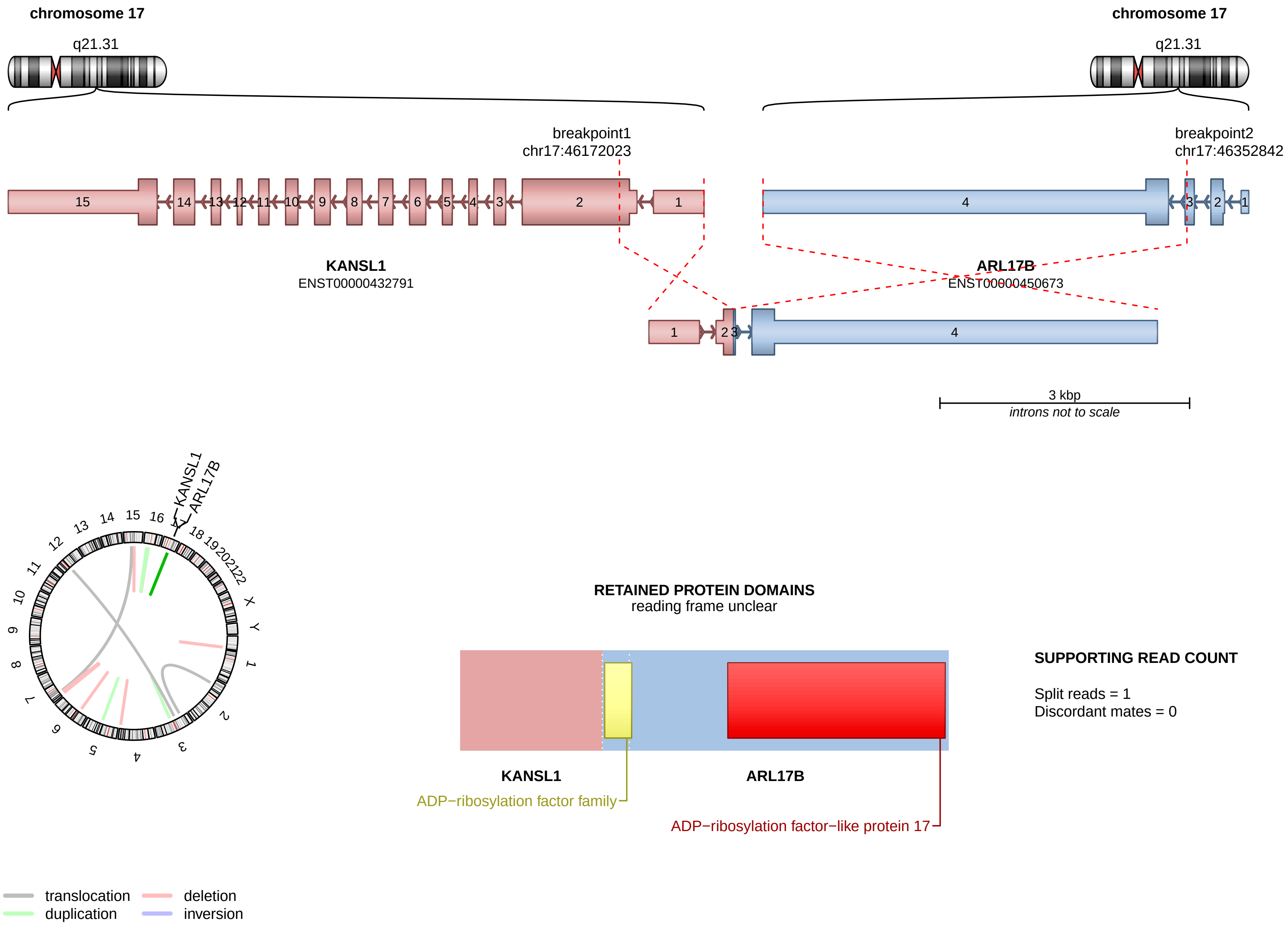
chromosome 17
q21.31
chromosome 17
q21.31
breakpoint1
chr17:46172023
breakpoint2
chr17:46352842
15
14
13
12
11
10
9
8
7
6
5
4
3
2
1
4
3
2
1
KANSL1
ENST00000432791
ARL17B
ENST00000450673
1
2
3
4
3 kbp
introns not to scale
KANSL1
ARL17B
RETAINED PROTEIN DOMAINS
reading frame unclear
SUPPORTING READ COUNT
Split reads = 1
Discordant mates = 0
KANSL1
ARL17B
ADP−ribosylation factor family
ADP−ribosylation factor−like protein 17
translocation
deletion
duplication
inversion

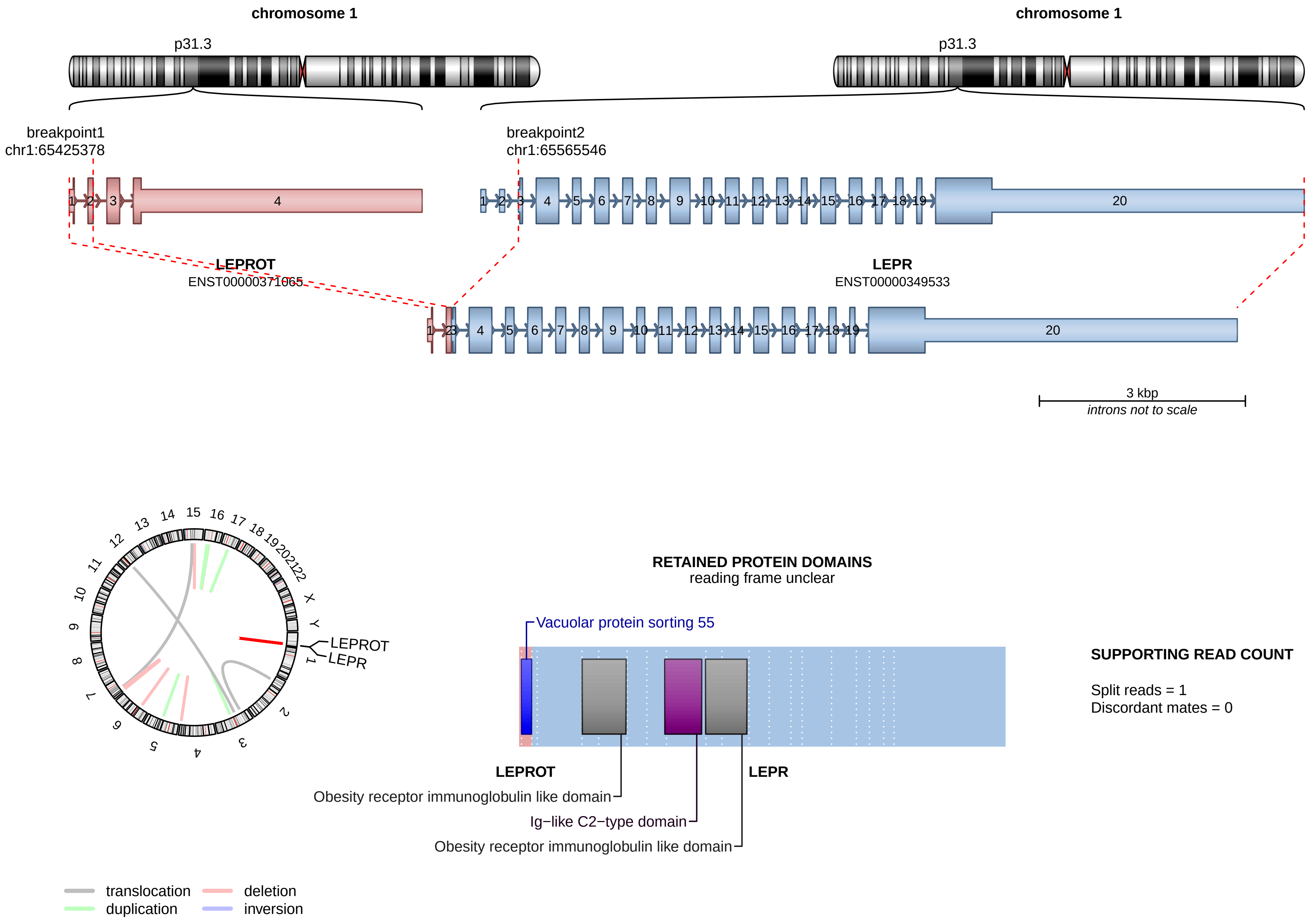

chromosome 1
p31.3
chromosome 1
p31.3
breakpoint1
chr1:65425378
breakpoint2
chr1:65565546
LEPROT
ENST00000371065
LEPR
ENST00000349533
3 kbp
introns not to scale
LEPROT
LEPR
translocation
duplication
deletion
inversion
RETAINED PROTEIN DOMAINS
reading frame unclear
Vacuolar protein sorting 55
LEPROT
LEPR
Obesity receptor immunoglobulin like domain
Ig−like C2−type domain
Obesity receptor immunoglobulin like domain
SUPPORTING READ COUNT
Split reads = 1
Discordant mates = 0

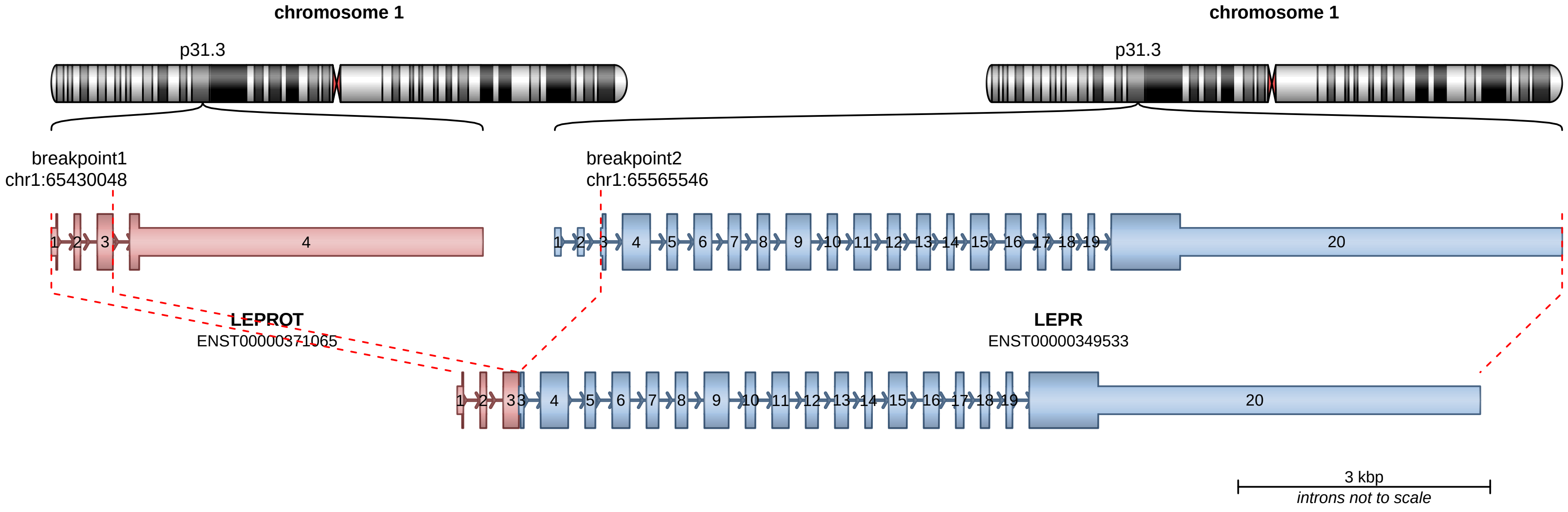

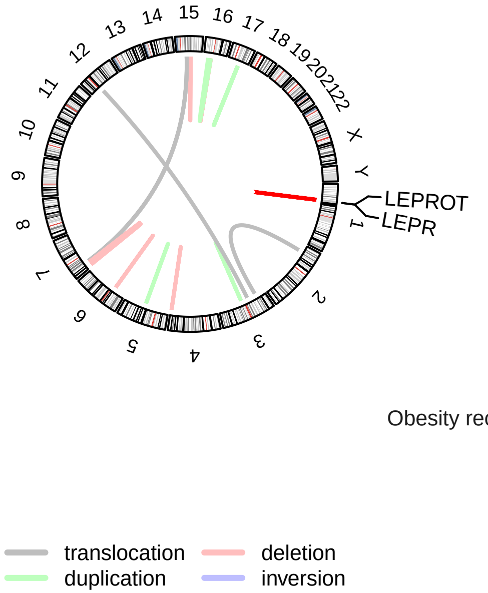

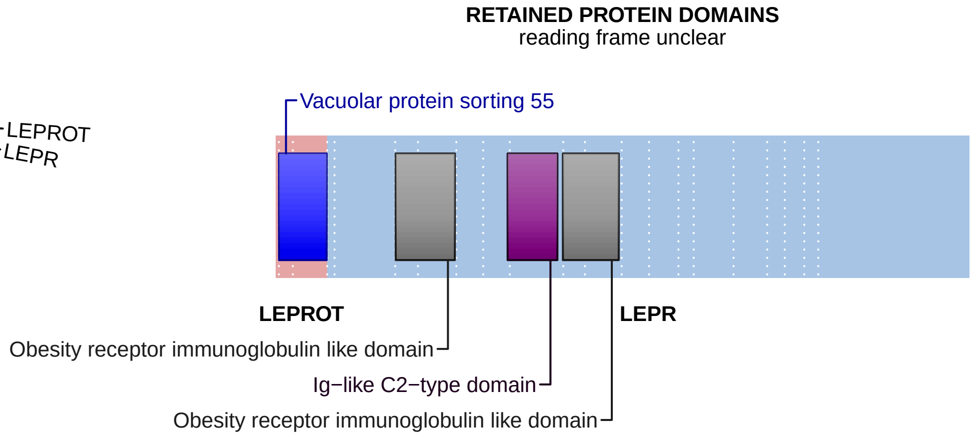

SUPPORTING READ COUNT

Split reads = 1
Discordant mates = 0

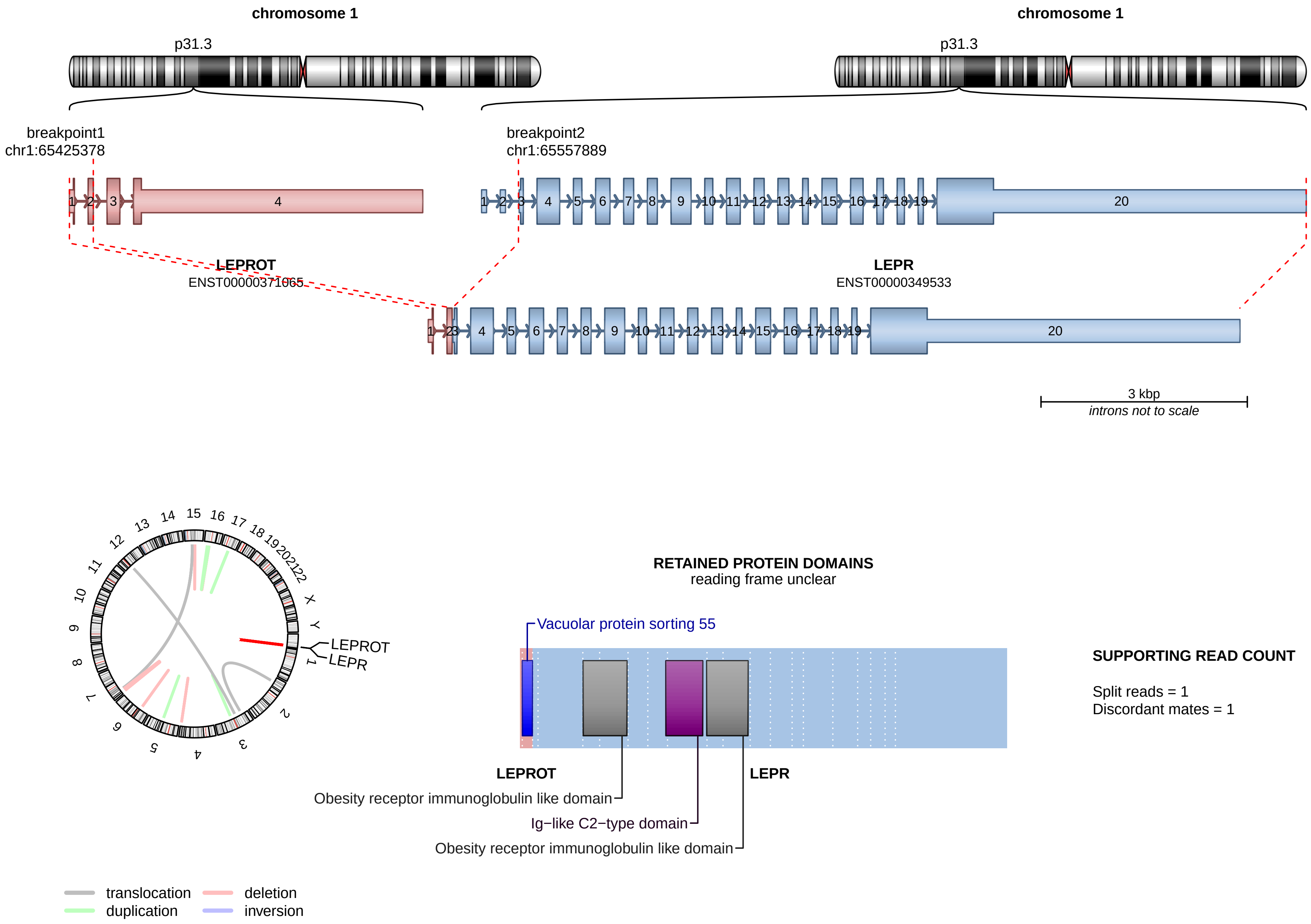
chromosome 1
p31.3
chromosome 1
p31.3
breakpoint1
chr1:65425378
breakpoint2
chr1:65557889
LEPROT
ENST00000371065
LEPR
ENST00000349533
3 kbp
introns not to scale
LEPROT
LEPR
RETAINED PROTEIN DOMAINS
reading frame unclear
Vacuolar protein sorting 55
LEPROT
LEPR
Obesity receptor immunoglobulin like domain
Ig−like C2−type domain
Obesity receptor immunoglobulin like domain
SUPPORTING READ COUNT
Split reads = 1
Discordant mates = 1
translocation
duplication
deletion
inversion

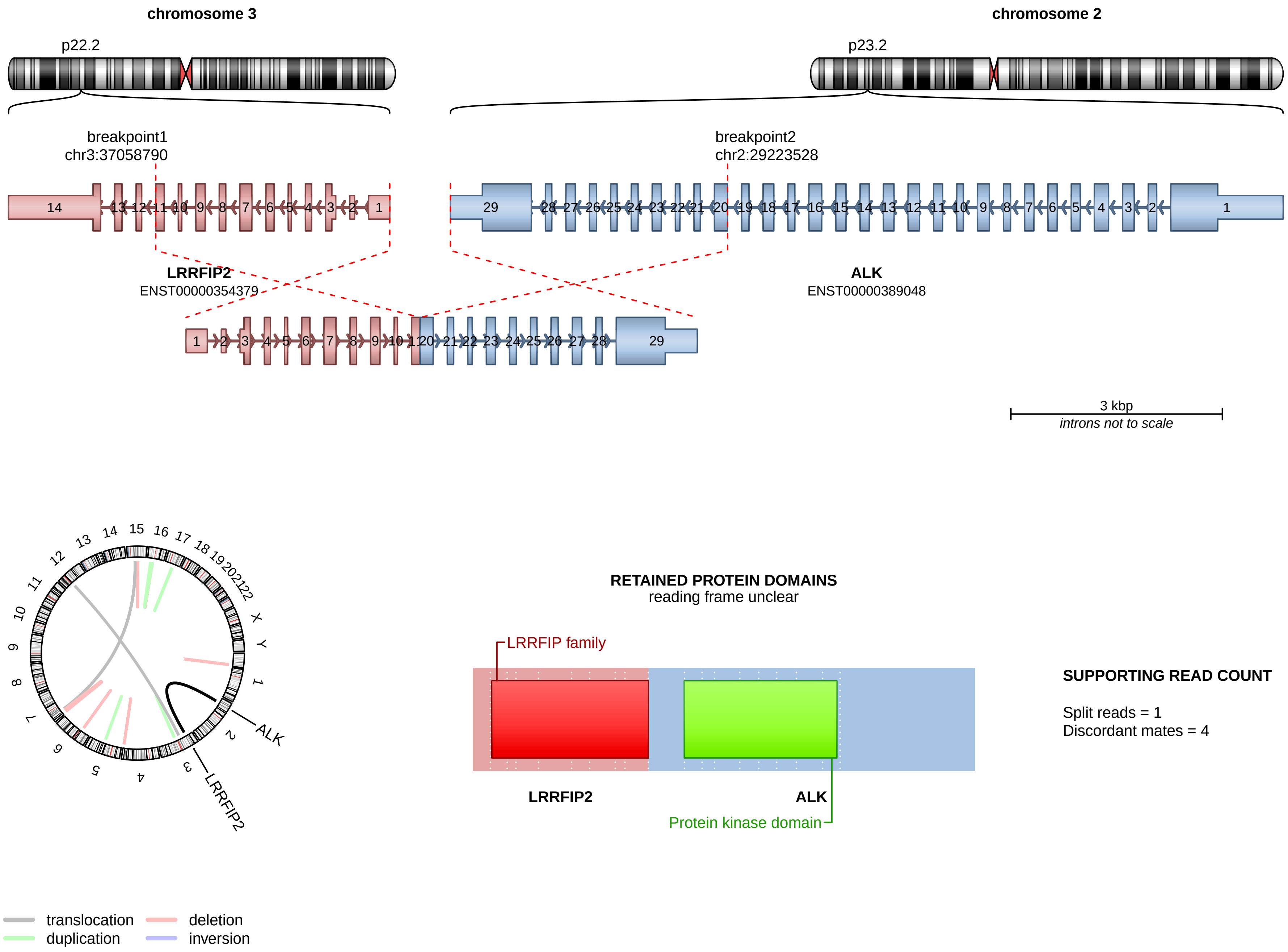
chromosome 3
p22.2
chromosome 2
p23.2
breakpoint1
chr3:37058790
breakpoint2
chr2:29223528
LRRFIP2
ENST00000354379
ALK
ENST00000389048
3 kbp
introns not to scale
RETAINED PROTEIN DOMAINS
reading frame unclear
LRRFIP family
LRRFIP2
ALK
Protein kinase domain
SUPPORTING READ COUNT
Split reads = 1
Discordant mates = 4
translocation
deletion
duplication
inversion

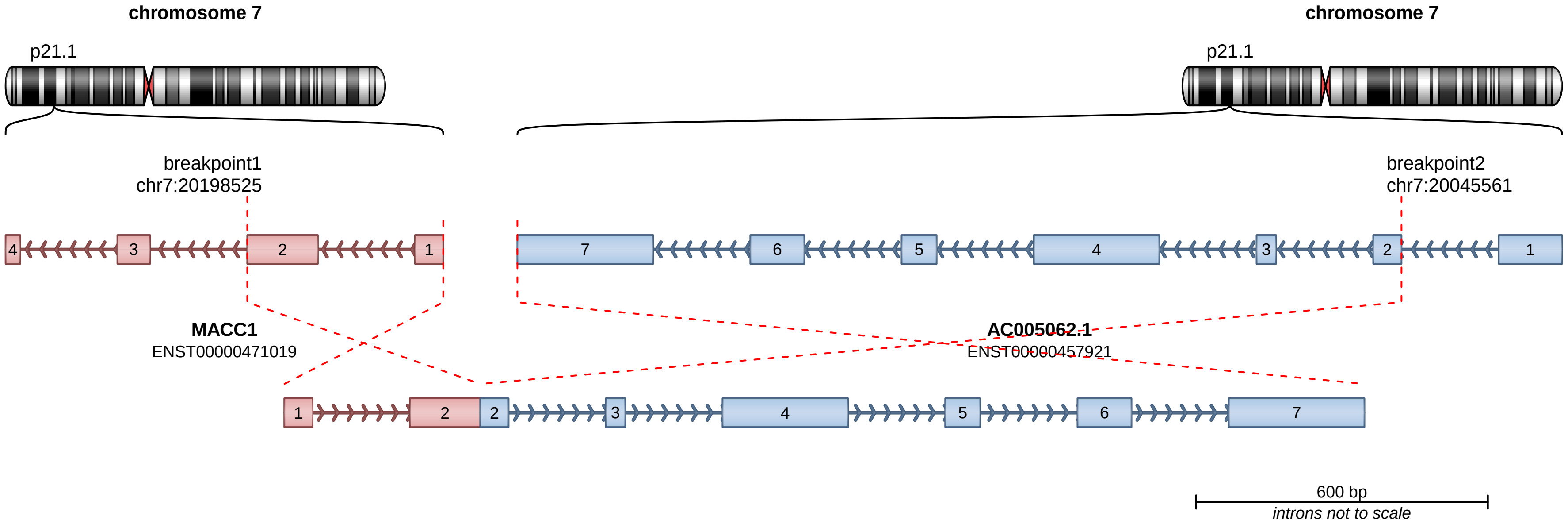

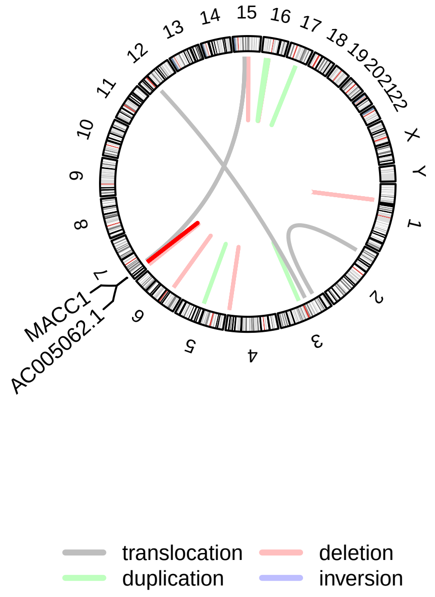

Genes are not protein−coding.

SUPPORTING READ COUNT

Split reads = 1
Discordant mates = 0

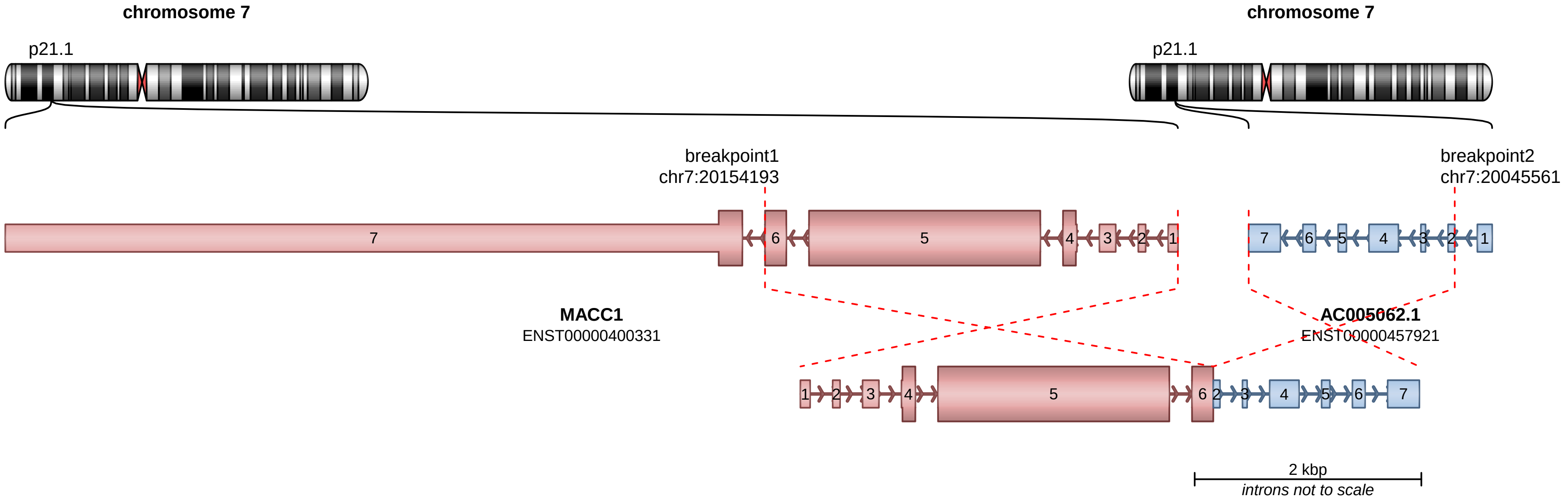

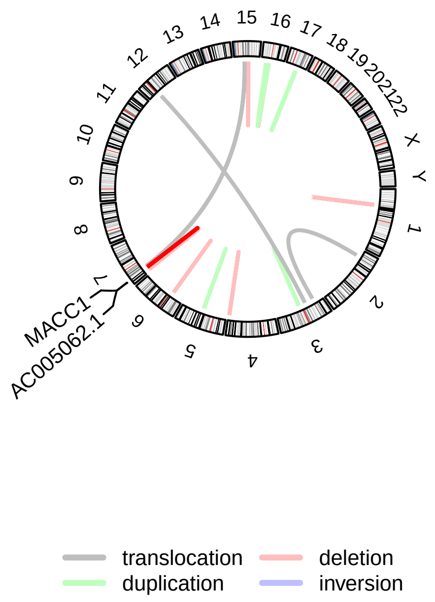

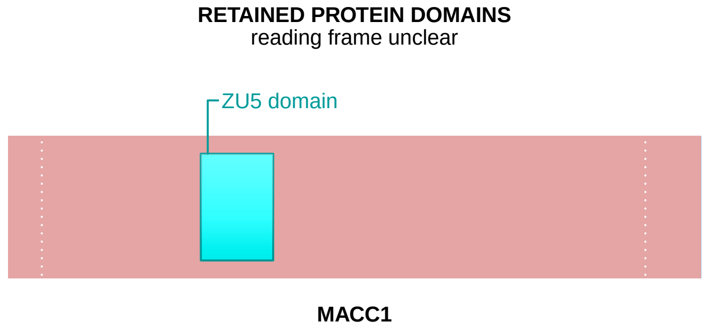

SUPPORTING READ COUNT

Split reads = 1
Discordant mates = 0

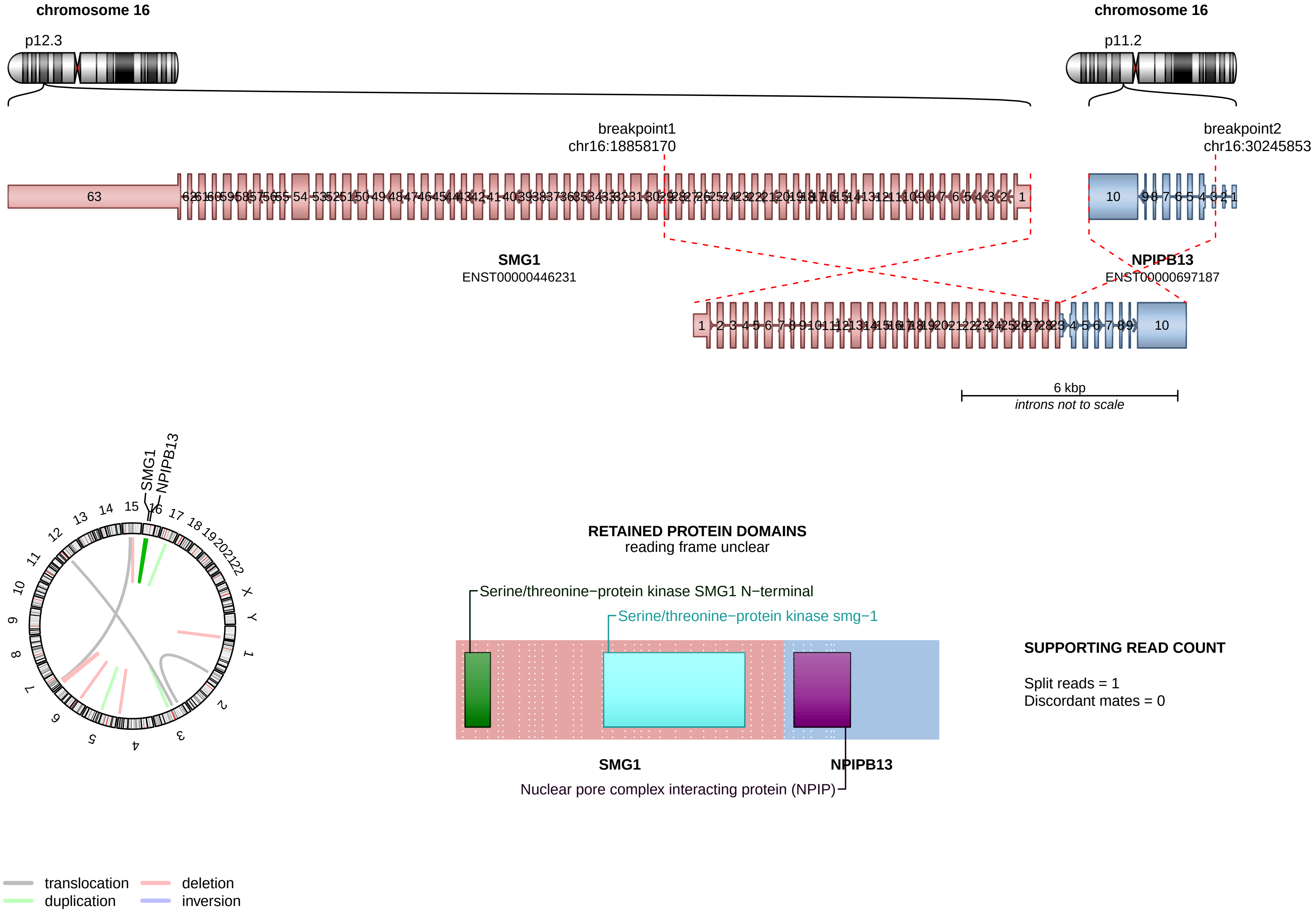
chromosome 16
p12.3
chromosome 16
p11.2
breakpoint1
chr16:18858170
breakpoint2
chr16:30245853
63
SMG1
ENST00000446231
NPIPB13
ENST00000697187
10
6 kbp
introns not to scale
SMG1
NPIPB13
RETAINED PROTEIN DOMAINS
reading frame unclear
Serine/threonine−protein kinase SMG1 N−terminal
Serine/threonine−protein kinase smg−1
SMG1
NPIPB13
Nuclear pore complex interacting protein (NPIP)
SUPPORTING READ COUNT
Split reads = 1
Discordant mates = 0
translocation
deletion
duplication
inversion

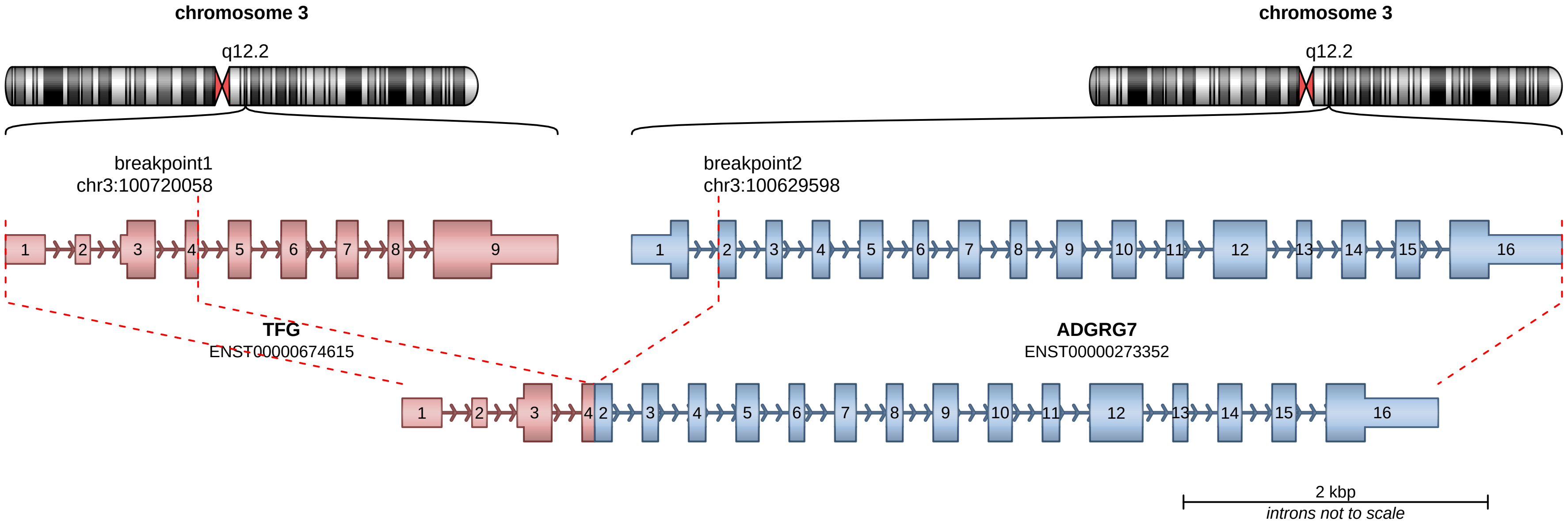

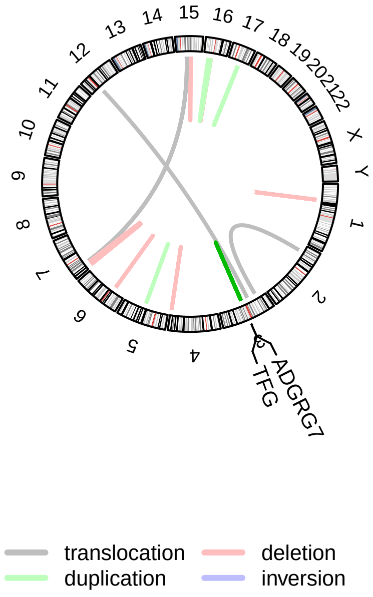

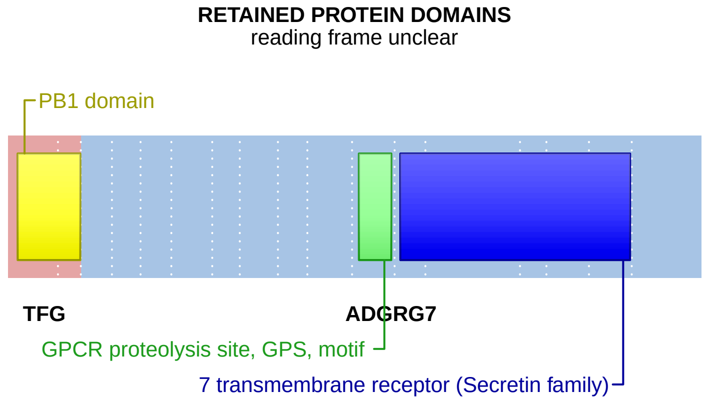

SUPPORTING READ COUNT

Split reads = 6
Discordant mates = 11

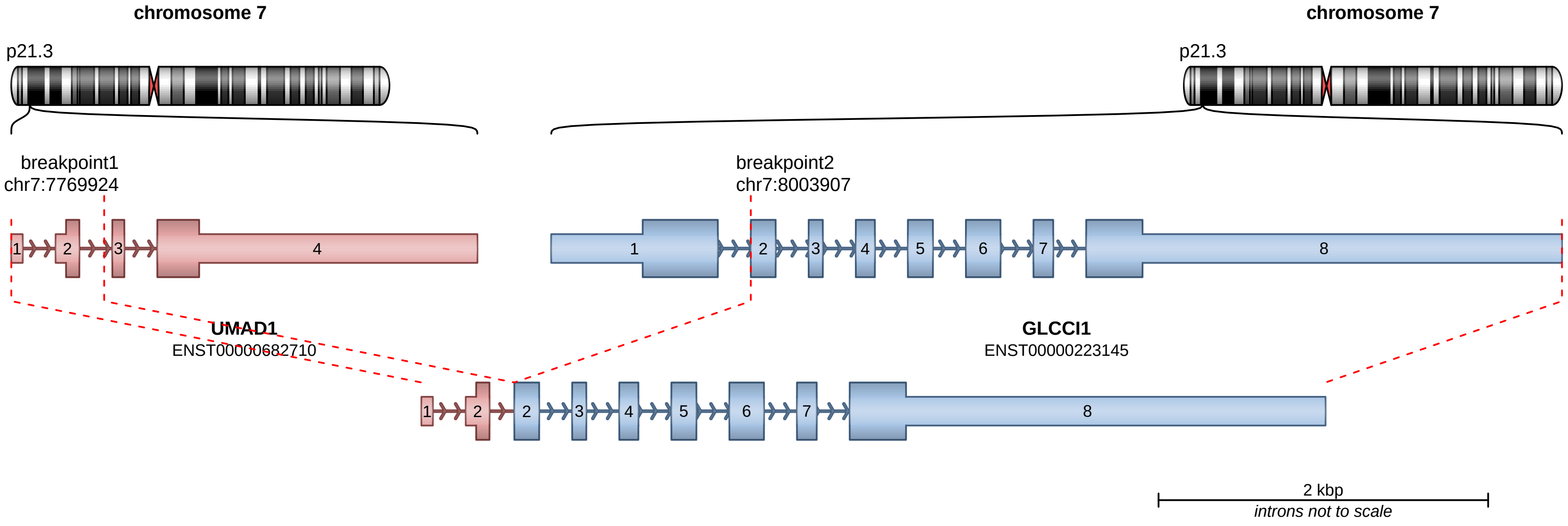

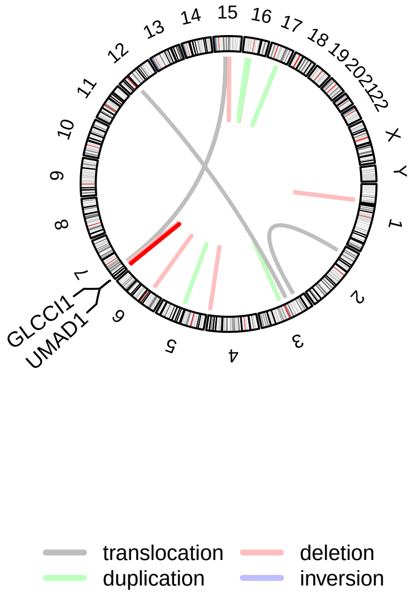

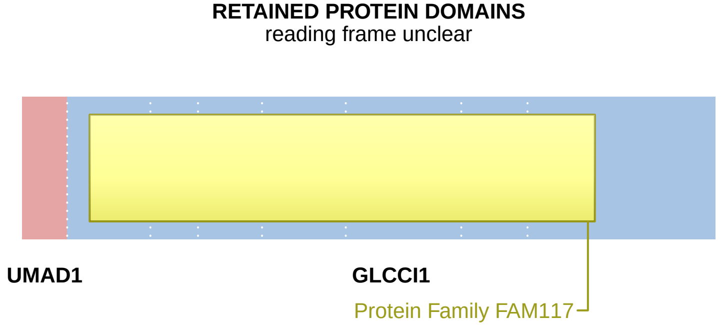

SUPPORTING READ COUNT

Split reads = 1
Discordant mates = 0

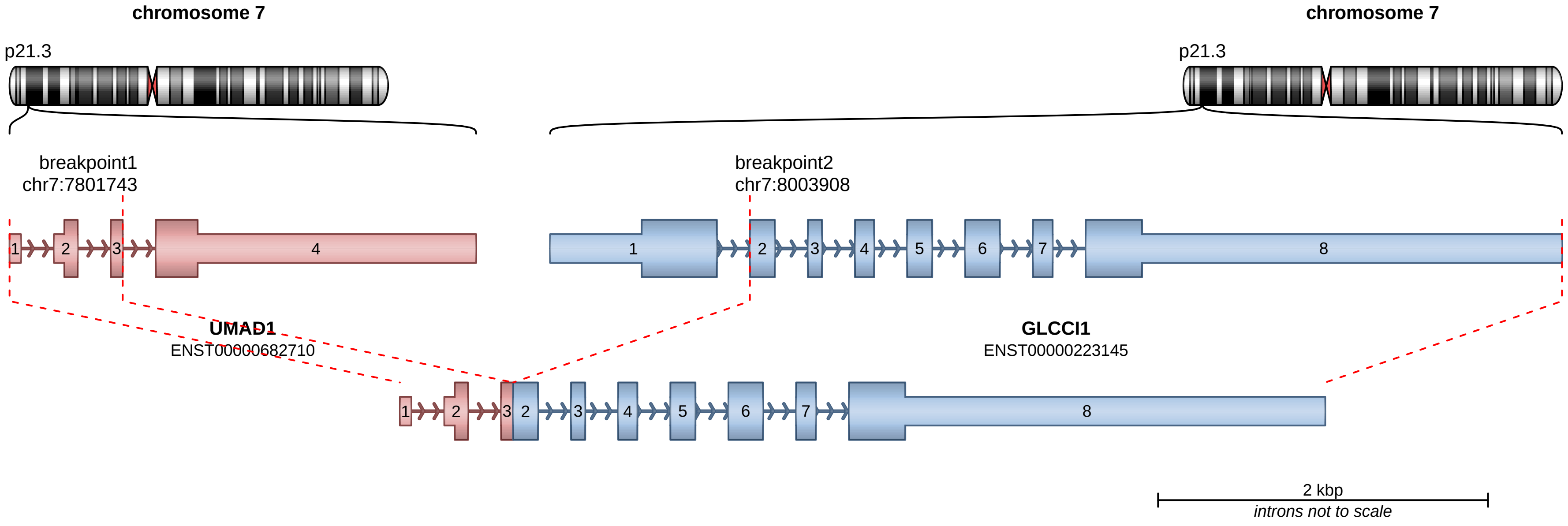

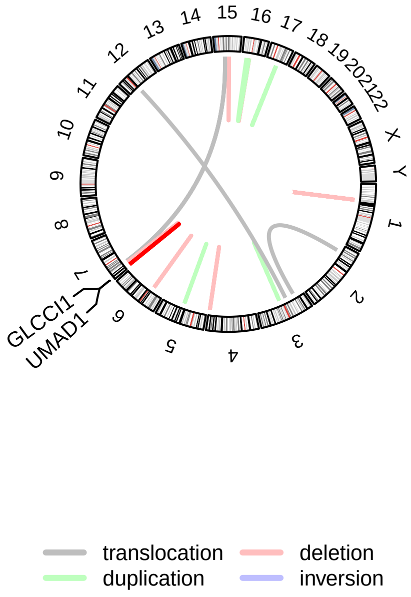

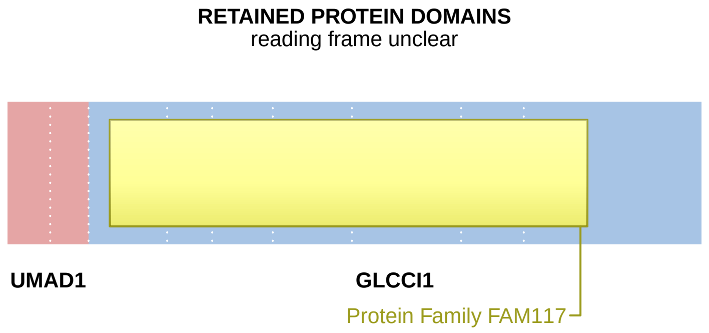

SUPPORTING READ COUNT

Split reads = 2
Discordant mates = 1

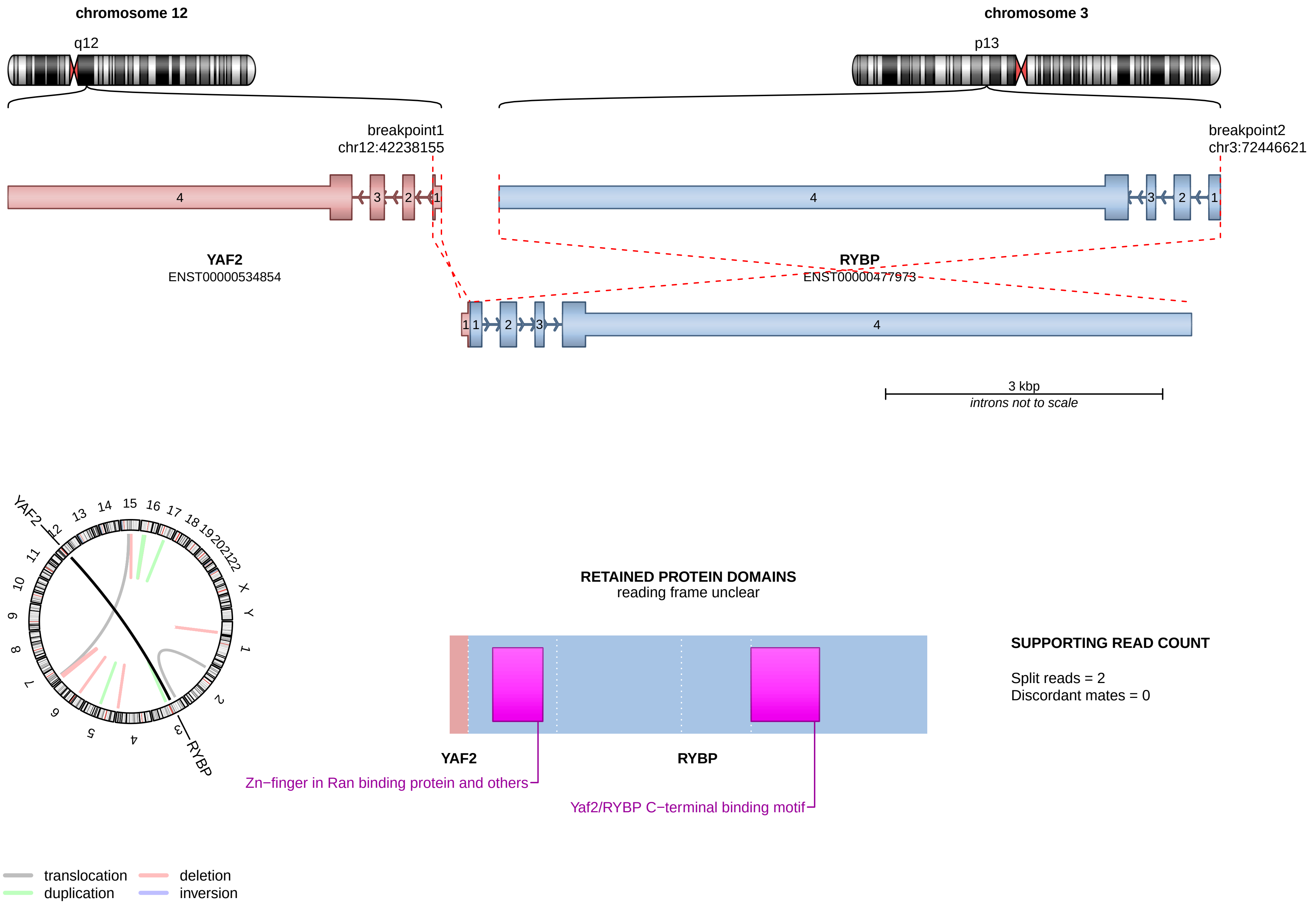
chromosome 12
q12
chromosome 3
p13
breakpoint1
chr12:42238155
breakpoint2
chr3:72446621
YAF2
ENST00000534854
RYBP
ENST00000477973
3 kbp
introns not to scale
YAF2
RYBP
RETAINED PROTEIN DOMAINS
reading frame unclear
YAF2
RYBP
Zn−finger in Ran binding protein and others
Yaf2/RYBP C−terminal binding motif
SUPPORTING READ COUNT
Split reads = 2
Discordant mates = 0
translocation
deletion
duplication
inversion
