# Supplementary figures and images for: Detection of fusion events by RNA sequencing in FFPE versus freshly frozen colorectal cancer tissue samples
Source: Front Mol Biosci. 2025 Jan 21;11:1448792. doi: 10.3389/fmolb.2024.1448792 (PMC11791353; doi:10.3389/fmolb.2024.1448792)

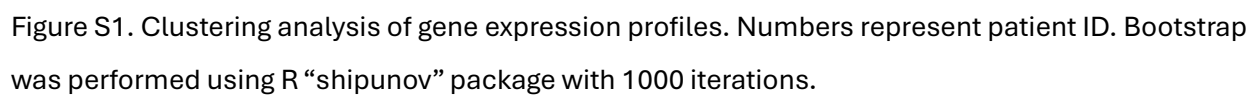

Supplement: Supplementary file 4 [file Image1.pdf]
